# Ferroptosis in Recurrent Vulvovaginal Candidiasis Through Integrated Bioinformatics and Experimental Validation

**DOI:** 10.3390/antiox15040407

**Published:** 2026-03-24

**Authors:** Yue-Min Hou, Hui Yu, Fang Feng, Hao-Yan Yao, Jin-Meng Yao, Rui-Fang An

**Affiliations:** 1 Department of Gynecology and Obstetrics, The Second Affiliated Hospital of Xi ‘an Jiaotong University, No.157 Siwu Road, Xi’an 710004, China; 18896500386@163.com; 2 Cancer Biology Research Center (Key Laboratory of the Ministry of Education), Tongji Hospital, Tongji Medical College, Huazhong University of Science and Technology, Wuhan 430074, China; yuhui_xjtu@163.com; 3 National Clinical Research Center for Obstetrics and Gynecology, Department of Gynecological Oncology, Tongji Hospital, Tongji Medical College, Huazhong University of Science and Technology, Wuhan 430074, China; 4 Department of Gynecology and Obstetrics, The First Affiliated Hospital of Xi ‘an Jiaotong University, No.157 Siwu Road, Xi’an 710004, China; ff230520@163.com (F.F.); yaohaoyan0115@163.com (H.-Y.Y.); yaojinmeng1571@163.com (J.-M.Y.)

**Keywords:** bioinformatics, ferroptosis, recurrent vulvovaginal candidiasis (RVVC), Gene Expression Omnibus (GEO), Ferrostatin-1 (Fer-1), macrophage

## Abstract

Background: Recurrent vulvovaginal candidiasis (RVVC) is a chronic inflammatory disease primarily caused by *Candida albicans* (*C. albicans*). Its pathogenesis remains incompletely understood, and clinical management is challenged by recurrence and drug resistance. Ferroptosis, an iron-dependent form of programmed cell death driven by lipid peroxidation, has been implicated in various infectious and inflammatory diseases. However, its role in RVVC remains unclear, with a particular lack of evidence from clinical samples and animal experiments. Objective: This study aimed to investigate the association between RVVC and ferroptosis. First, we analyzed high-throughput sequencing data from human RVVC samples in the Gene Expression Omnibus (GEO) database to identify the expression profile of ferroptosis-related genes. Second, using an established murine model of chronic vulvovaginal candidiasis (CVVC), we validated changes in ferroptosis-related markers in vaginal tissues in vivo. Furthermore, an in vitro model of *C. albicans*-infected bone marrow-derived macrophages (BMDMs) was employed to explore the underlying mechanisms. This study provides experimental evidence for elucidating the pathogenesis of RVVC and exploring novel therapeutic strategies. Methods: The RVVC-related gene expression dataset GSE278036 was obtained from the GEO database. Differentially expressed genes (DEGs) were screened using the DESeq2 algorithm and intersected with ferroptosis-related genes from the FerrDb database to identify key targets. A protein–protein interaction (PPI) network was constructed using the STRING database and Cytoscape software, and hub genes were identified via the Betweenness centrality algorithm. Functional and pathway analyses, including gene set enrichment analysis (GSEA), Gene Ontology (GO), Kyoto Encyclopedia of Genes and Genomes (KEGG), and WikiPathways, were performed. Immune infiltration analysis characterized the immune microenvironment in RVVC patients. A CVVC mouse model was established in vivo, and a *C. albicans*-BMDMs infection model was established in vitro. The ferroptosis inhibitor ferrostatin-1 (Fer-1) was administered to investigate the pathological function and regulatory mechanisms of ferroptosis in RVVC at the molecular, cellular, and tissue levels. Results: Differential analysis identified 3132 DEGs in RVVC, which intersected with ferroptosis-related genes to yield 194 key targets. Among them, 20 hub genes were identified, including ferroptosis regulators and inflammatory factors. Functional enrichment analysis confirmed that these shared targets regulate RVVC pathology through a “ferroptosis-inflammation-immunity” multi-pathway network. Immune infiltration analysis revealed a specific immune disorder in RVVC patients characterized by “activation of the pro-inflammatory innate immune axis and suppression of the adaptive immune axis,” which was closely associated with ferroptosis-related genes. In vivo and in vitro experiments confirmed that *C. albicans* infection induced ferroptosis in vaginal tissues and macrophages, as manifested by lipid ROS accumulation, Fe^2+^ overload, GSH depletion, downregulation of GPX4 and SLC7A11, upregulation of ACSL4, 4-HNE, and MDA, and mitochondrial structural damage. Macrophages were identified as key target cells for ferroptosis, and their ferroptosis led to impaired antifungal function. Fer-1 treatment significantly inhibited ferroptosis, reduced vaginal histopathological damage and inflammatory cell infiltration, decreased fungal burden, downregulated abnormally elevated inflammatory factors, and restored Th1/Th2 immune balance. Furthermore, Fer-1 preserved macrophage viability and enhanced their antifungal killing capacity. Conclusions: This study provides the first evidence linking RVVC to ferroptosis through a combination of clinical data analysis and experiments, suggesting that ferroptosis is involved in its pathological process. These findings offer a new perspective for elucidating RVVC pathogenesis and developing targeted therapeutic strategies.

## 1. Introduction

Vulvovaginal candidiasis (VVC) is an opportunistic mucocutaneous infection of the vulvovaginal tract caused by *Candida* species, with *Candida albicans* (*C. albicans*), accounting for approximately 90% of cases [1,2]. Epidemiological data indicate that about 75% of women experience at least one episode of VVC during their lifetime, and 5–9% of these develop recurrent VVC (RVVC), defined as three or more symptomatic episodes per year [3]. Although not directly life-threatening, RVVC imposes a significant physical, psychological, and economic burden, and has been associated with anxiety and depression [4]. Current research primarily focuses on immune dysregulation, biofilm formation, and drug resistance; however, these factors alone cannot fully explain the recurrence of RVVC [5,6]. This suggests that our understanding of the core molecular pathways driving its chronic inflammation and tissue damage remains incomplete, highlighting an urgent need to explore novel pathophysiological mechanisms.

Ferroptosis, an iron-dependent form of regulated cell death driven by lipid peroxidation, has emerged as a mechanistic framework for tissue injury in infectious and inflammatory diseases [7]. Unlike apoptosis or necrosis, ferroptosis is characterized by iron-catalyzed accumulation of lipid ROS and failure of antioxidant defense, particularly the glutathione (GSH)–GPX4 axis [8]. Increasing evidence indicates that pathogens can reprogram host iron metabolism and redox homeostasis to modulate ferroptosis and thereby influence infection outcomes. For instance, *Mycobacterium tuberculosis* induces ferroptosis in macrophages to compromise cell survival [9], whereas *Helicobacter pylori* upregulates GPX4 to restrain ferroptosis in gastric epithelial cells and facilitate colonization [10]. Notably, *C. albicans* infection triggers marked oxidative stress, a well-recognized upstream driver of ferroptosis [11]. It can disrupt host iron homeostasis and promote excessive ROS generation, thereby accelerating lipid peroxidation in infected mucosal tissues [11,12]. Perturbation of iron availability may support fungal persistence while simultaneously creating a pro-oxidative microenvironment permissive for ferroptotic injury [13]. In the vaginal niche, epithelial cells and resident immune cells are repeatedly exposed to microbial and inflammatory stimuli, exhibit high metabolic activity, and depend on tightly controlled iron and redox homeostasis to maintain mucosal defense [14,15]. These characteristics suggest that persistent *C. albicans*-associated oxidative stress could render local vaginal cells particularly vulnerable to ferroptosis. This observation led us to propose a scientific hypothesis: in RVVC, infection may induce ferroptosis in local vaginal tissues, thereby promoting excessive inflammation, cell death, and tissue damage, ultimately contributing to disease recurrence. However, direct evidence from clinical data and in vivo experiments supporting this hypothesis is currently lacking.

The increasing availability of high-throughput transcriptomic data enables systematic interrogation of disease-relevant pathways through bioinformatics. Analysis of RVVC-related datasets in the Gene Expression Omnibus (GEO) can reveal ferroptosis-associated gene signatures and their potential immune contexture in patients [16]. In parallel, the chronic vulvovaginal candidiasis (CVVC) mouse model, which recapitulates persistent vaginal *C. albicans* colonization and chronic inflammatory responses, provides an experimental platform to validate ferroptosis involvement in vivo [17].

Based on this rationale, the present study aims to investigate the role of ferroptosis in RVVC by integrating bioinformatics analysis, animal model validation, and in vitro experiments. First, we mined transcriptomic data from RVVC patients in the GEO database to construct expression and regulatory networks of ferroptosis-related genes. Second, using a CVVC mouse model, we will be using alterations in key ferroptosis markers in vivo and evaluating the therapeutic potential of a ferroptosis inhibitor. Finally, an in vitro model of *C. albicans*-infected macrophages will be used to further verify the ability of the fungus to induce ferroptosis. This study is expected to provide a novel theoretical foundation for elucidating the recurrence mechanism of RVVC and for developing new therapeutic strategies targeting ferroptosis.

## 2. Materials and Methods

### 2.1. Ethical Statement

All animal experiments were conducted in accordance with the International Guiding Principles for Biomedical Research Involving Animals and approved by the Animal Ethics Committee of Xi’an Jiaotong University (Approval No. XJTUAE2025-3380).

### 2.2. Data Acquisition and Differential Expression Analysis for RVVC

The gene expression profile dataset GSE278036 (healthy controls (CTRL), n = 18; RVVC, n = 19; platform GPL34284) related to RVVC was downloaded from the GEO database (https://www.ncbi.nlm.nih.gov/geo/; accessed on 9 September 2025) [16]. Differential expression analysis was performed using the DESeq2 package (version 1.38.3) in R software. Genes with an adjusted *p*-value (adj.*p*) < 0.05 and an absolute log2 fold change (|log_2_FC|) > 0.58 were identified as differentially expressed genes (DEGs) between the RVVC group and the healthy control group.

### 2.3. Screening of Ferroptosis-Related Genes and Intersection Analysis

Literature-validated ferroptosis-related genes, including drivers, suppressors, unclassified and markers (1291 genes in total, see as Table S1), were obtained from the FerrDb database (http://www.zhounan.org/ferrdb/; accessed on 1 October 2025). This gene set was intersected with the RVVC DEGs to identify key targets shared by RVVC and ferroptosis. A Venn diagram was generated for visualization using the VennDiagram package (version 1.7.3) in R.

### 2.4. Protein–Protein Interaction Network Construction and Hub Gene Identification

The list of intersecting genes was uploaded to the STRING database (version 12.0, https://string-db.org/; accessed on 1 October 2025) to construct a protein–protein interaction (PPI) network, with a minimum interaction confidence score set to >0.4. The resulting network was imported into Cytoscape software (version 3.9.1) for visualization. The CytoHubba plugin was used with the “Betweenness” centrality algorithm to rank nodes, and the top 20 hub genes were selected [18].

### 2.5. Functional and Pathway Enrichment Analysis

Gene Ontology (GO) functional annotation (including biological process, molecular function, and cellular component) [19] and Kyoto Encyclopedia of Genes and Genomes (KEGG) pathway [20] enrichment analysis of the intersecting genes were performed using the STRING database [21]. Complementary pathway analysis was additionally conducted using the WikiPathways database (https://www.wikipathways.org/; accessed on 15 October 2025) [22]. Gene Set Enrichment Analysis (GSEA) was performed using the R package clusterProfiler (version 4.6.2) to evaluate the enrichment trend of ferroptosis pathways in the overall RVVC differential expression profile [23]. The significance threshold for all enrichment results was set at an adj.*p* < 0.05.

### 2.6. Immune Infiltration Analysis

Based on the gene expression matrix of RVVC and control samples, the relative proportions of 22 immune cell subsets were estimated using the CIBERSORT algorithm (https://cibersortx.stanford.edu/; accessed on 2 November2025) [24]. Differences in immune cell abundance between the two groups were compared. A correlation network among immune cells was analyzed using Spearman’s rank correlation coefficient. Further, the above analysis was conducted on the ferroptosis genes related to RVVC.

### 2.7. Animals

Female Balb/c mice (6–8 weeks old, weighing 18–25 g) were purchased from the Experimental Animal Center of Xi’an Jiaotong University. All mice were healthy prior to experiments and housed in a specific pathogen-free (SPF) environment with free access to food and water (5 mice per cage). All experimental procedures were performed in accordance with relevant animal ethics regulations.

### 2.8. C. albicans Strain

*C. albicans* (ATCC 10231) was kindly provided by Prof. Li Zheng’s group at Northwest University and stored at −80 °C in a cryopreservation solution containing 25% glycerol. Prior to each experiment, *C. albicans* was streaked onto Sabouraud Dextrose Agar (SDA) plates and incubated at 35 °C for 36 h. Single colonies were then picked for experiments to ensure optimal growth characteristics and purity.

### 2.9. Preparation of C. albicans Suspension

A small amount of the glycerol-preserved *C. albicans* stock was spread on an Sabouraud’s dextrose agar (SDA) plate and incubated at 35 °C. After white, round colonies appeared, a single colony was picked and streaked onto a fresh SDA plate for further incubation under the same conditions. Subsequently, a single colony was inoculated into Sabouraud’s dextrose broth (SDB) and cultured in a shaker incubator at 35 °C and 180 rpm for 12 h. The activated culture was collected, washed once with phosphate-buffered saline (PBS), and resuspended in PBS at a concentration of 1.0 × 10^8^ colony-forming units (CFU)/mL for use.

### 2.10. Establishment of the CVVC Mouse Model

In this study, a CVVC model was employed as an experimental surrogate for human RVVC, as it recapitulates key pathological features observed in RVVC patients, including persistent fungal colonization, epithelial damage, and chronic immune dysregulation. The CVVC mouse model was established with minor modifications based on published protocols [17]. Mice were randomly divided into three groups: Control group, *C. albicans* group, and *C. albicans* + ferrostatin-1 (Fer-1) group. To induce a pseudo-estrus state conducive to infection, all mice received subcutaneous injections of estradiol benzoate (0.2 mg per mouse daily, administered as 0.1 mg twice daily via abdominal midline subcutaneous injection) for 7 days prior to infection. This injection was repeated every other day throughout the experiment to maintain susceptibility.

Mice in the infection groups were inoculated intravaginally with 20 μL of the *C. albicans* suspension (1.0 × 10^8^ CFU/mL) using a micropipette, while control mice received an equal volume of PBS. Mice were held in a head-down position for 5 min post-inoculation to prevent leakage. Inoculation was performed daily. On day 5 post-infection, vaginal lavage was performed three times with 40 μL PBS per wash. The lavage fluid was collected, serially diluted, and examined microscopically for the presence of yeast and hyphae. Additionally, 20 μL of the lavage fluid was plated on CHROMagar™ Candida plates and SDA plates, followed by incubation at 35 °C for 48 h for fungal identification. Vaginal tissue from infected mice was also collected for Grocott’s methenamine silver (GMS) staining. Successful model establishment was confirmed by the presence of clinical signs (vulvovaginal redness, swelling, exudate) and positive fungal culture/microscopy (Supplement Figure S1)

Starting from day 7 post-infection, mice in the Fer-1 treatment group received daily intraperitoneal injections of Fer-1 at 10 mg/kg for 7 days. The *C. albicans* group received equal-volume PBS injections, while the control group received no treatment.

### 2.11. Isolation, Culture, and Characterization of Bone Marrow-Derived Macrophages (BMDMs)

BMDMs were isolated from the femurs and tibias of euthanized mice. Bones were flushed with PBS containing 1% penicillin-streptomycin. The collected cell suspension was filtered through a 70 μm cell strainer, centrifuged, and treated with red blood cell lysis buffer. After washing, cells were resuspended in complete DMEM (Corning Incorporated, Corning, NY, USA) supplemented with 20 ng/mL macrophage colony-stimulating factor (M-CSF, MCE, Monmouth Junction, NJ, USA) and seeded in culture dishes. The medium was partially replaced on day 3, and BMDMs were harvested on day 7 for experiments.

For flow cytometry, harvested BMDMs were stained with FITC-conjugated anti-CD11b and APC-conjugated anti-F4/80 antibodies and analyzed (Thermo Fisher Scientific, Waltham, MA, USA). For immunofluorescence, cells were fixed, permeabilized, blocked, and incubated with an anti-F4/80 primary antibody overnight at 4 °C, followed by a FITC-conjugated secondary antibody and DAPI nuclear staining for observation under a fluorescence microscope (Thermo Fisher Scientific, Waltham, MA, USA).

### 2.12. In Vitro Co-Culture of BMDMs with C. albicans

BMDMs were co-cultured with *C. albicans* at a multiplicity of infection (MOI) of 1:1 for 10 h for subsequent experiments. The experimental groups included: control BMDMs, *C. albicans*-infected BMDMs, and *C. albicans*-infected BMDMs pretreated with Fer-1. For the Fer-1 pretreatment group, BMDMs were incubated with 15 μM Fer-1 for 6 h prior to *C. albicans* infection.

### 2.13. Assessment of Fungal Burden

Vaginal lavage was performed as described in Section 2.10. The lavage fluid was serially diluted, plated on SDA plates, and incubated at 35 °C for 36 h before counting fungal colonies.

### 2.14. Macrophage Killing Assay

BMDMs were co-cultured with *C. albicans* (MOI 1:1) for 10 h, with or without Fer-1 pretreatment (as in Section 2.12). A control well containing an equal amount of *C. albicans* without BMDMs was included. After co-culture, cells were lysed with 0.02% Triton X-100 on ice for 5 min. The lysates were collected, centrifuged, resuspended, plated on SDA, and incubated for 36 h at 35 °C for colony counting. The fungal killing capacity was calculated.

### 2.15. Hematoxylin and Eosin (H&E) Staining

Formalin-fixed, paraffin-embedded vaginal tissues were sectioned at 4 μm thickness and stained with H&E (Beyotime Biotechnology Co., Ltd., Shanghai, China). Tissue morphology, mucosal integrity, inflammatory cell infiltration, and squamous epithelial structure were evaluated under a light microscope (Nikon Instruments Inc., Melville, NY, USA).

### 2.16. Immunohistochemistry (IHC) Staining

Tissue sections underwent antigen retrieval, blocking, and overnight incubation at 4 °C with primary antibodies against 4-hydroxynonenal (4-HNE, Abways, Shanghai, China) (1:200) or glutathione peroxidase 4 (GPX4, Abways, Shanghai, China) (1:200). After washing, HRP-conjugated secondary antibodies (Abways, Shanghai, China) (1:500) were applied, followed by development with 3,3′-diaminobenzidine (DAB, Beyotime Biotechnology Co., Ltd., Shanghai, China) and counterstaining with hematoxylin (Beyotime Biotechnology Co., Ltd., Shanghai, China). Images were captured and analyzed using ImageJ software (version 1.8.0).

### 2.17. Immunofluorescence (IF) Staining

Frozen vaginal tissue sections (4 μm) of vaginal tissue were subjected to antigen retrieval and blocking. Sections were incubated overnight at 4 °C with a mixture of primary antibodies (F4/80 plus 4-HNE, GPX4, or SLC7A11) (Abways, Shanghai, China) (1:500). After washing, fluorescent secondary antibodies and DAPI (Thermo Fisher Scientific, Waltham, MA, USA) were applied. Images were acquired using a Leica TCS SP8 confocal microscope (Leica Microsystems GmbH, Wetzlar, Germany) and analyzed with Image J software (version 1.54p).

### 2.18. TUNEL Assay

Apoptosis in vaginal tissue sections was detected using an In Situ Cell Death Detection Kit (Thermo Fisher Scientific, Waltham, MA, USA) according to the manufacturer’s instructions. Sections were observed under a fluorescence microscope (Leica Microsystems GmbH, Wetzlar, Germany).

### 2.19. Measurement of Fe^2+^, Malondialdehyde (MDA), and Glutathione (GSH)

Concentrations of Fe^2+^, MDA, and GSH in vaginal tissue homogenates or BMDM lysates were determined using commercial assay kits according to the manufacturer’s instructions (Elabscience Biotechnology Co., Ltd., Wuhan, China). Absorbance was measured using a microplate reader (BioTek Instruments, Inc., Winooski, VT, USA).

### 2.20. Western Blot

Total protein was extracted from vaginal tissues or BMDMs using RIPA lysis buffer containing protease and phosphatase inhibitors (Beyotime Biotechnology Co., Ltd., Shanghai, China). Protein concentration was determined via a BCA assay. Proteins (30 μg per lane) (Beyotime Biotechnology Co., Ltd., Shanghai, China) were separated by SDS-PAGE (10–12% gel), transferred to PVDF membranes, and blocked. Membranes were incubated overnight at 4 °C with primary antibodies against ACSL4, GPX4, SLC7A11, or GAPDH (all at 1:200 dilution, Abways, Shanghai, China). After washing, HRP-conjugated secondary antibodies (1:5000, Abways, Shanghai, China) were applied. Protein bands were visualized using an enhanced chemiluminescence (ECL, Thermo Fisher Scientific, Waltham, MA, USA) detection system and quantified using ImageJ software with GAPDH (Abways, Shanghai, China) as the loading control.

### 2.21. Lipid ROS Detection

For vaginal tissue, single-cell suspensions were prepared via tissue digestion. For BMDMs, cells were used directly or after detachment. Cells were incubated with 10 μM BODIPY™ 581/591 C11 (Lipid ROS probe, Thermo Fisher Scientific, Waltham, MA, USA) at 37 °C for 30 min, washed, and analyzed by flow cytometry (Thermo Fisher Scientific, Waltham, MA, USA) or observed under a fluorescence microscope (Leica Microsystems GmbH, Wetzlar, Germany). Data were analyzed using FlowJo software (version 10.8.2, FlowJo LLC, Ashland, OR, USA).

### 2.22. Mitochondrial Membrane Potential (MMP) Assay

BMDMs were incubated with JC-1 staining (Beyotime Biotechnology Co., Ltd., Shanghai, China) working solution at 37 °C for 20 min, washed, and resuspended. The shift from red fluorescence (JC-1 aggregates, high MMP) to green fluorescence (JC-1 monomers, low MMP) was observed under a fluorescence microscope or quantified by flow cytometry.

### 2.23. Lactate Dehydrogenase (LDH) Release Assay

BMDMs were seeded in 96-well plates. After experimental treatments (infection ± Fer-1), the culture supernatant was collected. LDH release was measured using a commercial cytotoxicity detection kit according to the manufacturer’s instructions. Absorbance was read at 490 nm, and the LDH release rate was calculated (Beyotime Biotechnology Co., Ltd., Shanghai, China).

### 2.24. Transmission Electron Microscopy (TEM)

BMDMs were collected, fixed overnight at 4 °C in 2.5% glutaraldehyde, and post-fixed in 1% osmium tetroxide. After dehydration in a graded ethanol and acetone series, the cells were embedded, ultrathin-sectioned, and double-stained with uranyl acetate and lead citrate. Sections were examined and imaged using a transmission electron microscope(HT7800, Hitachi, Tokyo, Japan).

### 2.25. Cytokine Antibody Array

Vaginal tissue homogenates (from 3 mice per group) were analyzed using the AAM-INF-1 inflammatory cytokine array (RayBiotech, Inc., Norcross, GA, USA) according to the manufacturer’s instructions. Arrays were scanned, and data were analyzed using GeneSpring software (version 14.9). Differentially expressed proteins were prioritized based on fold-change thresholds (≤0.83 or ≥1.2), together with array software-derived *p* values (*p* < 0.05), which were used as descriptive criteria for hit selection rather than as definitive inferential statistics. Key cytokines identified in the array were subsequently validated in independent cohorts by ELISA.

### 2.26. Enzyme-Linked Immunosorbent Assay (ELISA)

Levels of TNF-α, IL-1β, IL-17, IFN-γ, IL-4, and IL-2 in vaginal lavage fluid were measured using commercial ELISA kits according to the manufacturer’s instructions. Absorbance at 450 nm was read, and concentrations were calculated based on standard curves (Elabscience Biotechnology Co., Ltd. Wuhan, China).

### 2.27. Statistical Analysis

Statistical analysis was performed using GraphPad Prism (version 10.0), SPSS (version 29.0), and R software (version 4.2.0). Data normality was assessed using the Shapiro–Wilk test. For comparisons between two groups with normal distribution, an unpaired two-tailed Student’s *t*-test was used. For comparisons among multiple groups with normal distribution and homogeneity of variance, one-way analysis of variance (ANOVA) was performed followed by Tukey’s post hoc test. If the data did not meet the assumptions of normality or equal variance, the non-parametric Mann–Whitney U test (for two groups) or Kruskal–Wallis test with Dunn’s post hoc test (for multiple groups) was applied. Data are presented as mean ± standard deviation (SD) for parametric tests or median with interquartile range (IQR) for non-parametric tests. For tissue-based in vivo endpoints, n = 5 mice per group were used. For vaginal lavage cytokines and *C. albicans* burden measured by ELISA, n = 10 mice per group were used due to higher expected variability. For cytokine antibody array screening, vaginal tissue homogenates, n = 3 mice per group were used. Statistical power (1 − β) reflects the probability of correctly rejecting the null hypothesis when a true effect exists. For the present study, sample sizes were selected based on prior experience with the model and consistency with similar published studies. Post hoc power analysis was conducted using G*Power (version 3.1.9.7) to confirm that the achieved power for representative primary endpoints exceeded the conventional threshold of 0.80. Figure 18 and the graphical abstract were initially drafted with the assistance of Gemini 3 based on detailed prompts written by the authors to visualize their own scientific ideas. The generated drafts were subsequently manually reviewed, corrected, and refined by the authors in Adobe Illustrator to ensure scientific accuracy and consistency with the manu-script. All other figures were generated from original research data using GraphPad Prism and were further arranged in Adobe Illustrator.

## 3. Results

### 3.1. Screening of RVVC-Associated Ferroptosis-Related Genes

**Figure 1:**
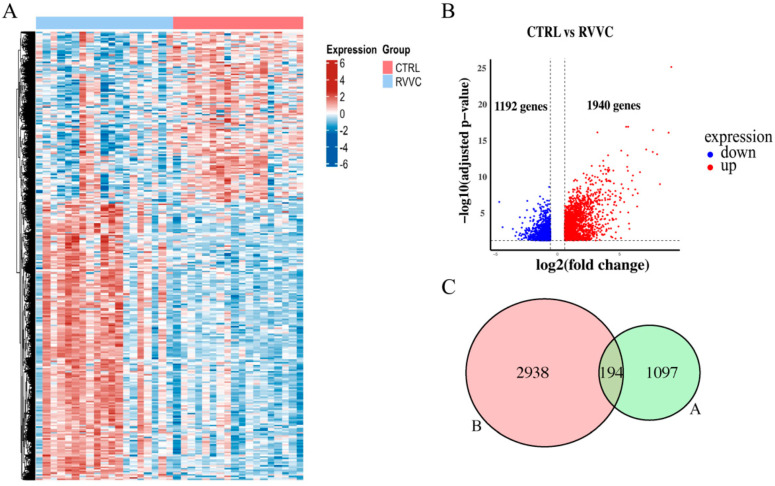
Identification of RVVC-associated ferroptosis-related genes. (**A**) DEGs between CTRL (n = 18) and RVVC patients (n = 19) in GSE278036. Red/blue indicates upregulated/downregulated expression (color intensity corresponds to normalized expression levels, range: −6 to 6). (**B**) Volcano plot of DEGs. Red/blue dots represent upregulated/downregulated genes (1940 up, 1192 down; −log_10_(adjusted *p*-value) reflects statistical significance, log_2_(fold change) reflects expression fold change). (**C**) Venn diagram illustrating the intersection between RVVC-associated DEGs (*n* = 3132) and ferroptosis-related genes (*n* = 1291) curated from the FerrDb database, yielding 194 overlapping candidate targets.

We obtained the RVVC-related gene expression dataset GSE278036 (CTRL, n = 18; RVVC, n = 19) from the GEO database. Differential expression analysis using the DESeq2 algorithm identified a total of 3132 DEGs, consisting of 1940 upregulated and 1192 downregulated genes (Figure 1A,B). Intersecting these DEGs with the 1291 ferroptosis-related genes from the FerrDb database yielded 194 key targets potentially shared between RVVC pathogenesis and ferroptosis regulation (Figure 1C). These intersecting genes may represent critical links connecting the pathological processes of RVVC with the regulatory mechanisms of ferroptosis.

### 3.2. Construction and Analysis of the PPI Network for RVVC and Ferroptosis Shared Targets

**Figure 2:**
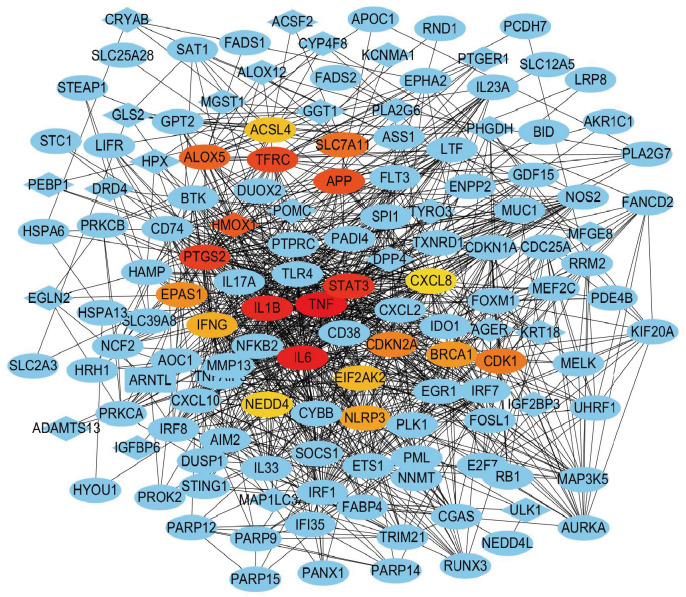
PPI network of RVVC-ferroptosis shared genes and hub genes visualization. PPI network. Nodes are labeled with gene symbols; the top 20 hub genes (ranked by Betweenness centrality are highlighted. In the network, the top 20 hub genes are highlighted using a color gradient from red to orange/yellow according to their ranking scores shown in the Table 1, with red indicating higher-ranked genes (higher scores) and orange/yellow indicating relatively lower-ranked genes (lower scores). Blue nodes represent the remaining non-hub genes.

To investigate the interactions among the 194 shared targets, we constructed a PPI network using the STRING 12.0 database and visualized it with Cytoscape software (version 3.1.9.7). The resulting network comprised 181 nodes and 111 interaction edges. To identify core regulatory genes within this network, we employed the Betweenness centrality algorithm for ranking and selected the top 20 hub genes (Table 1). These hub genes included TNF, IL6, IL1B, STAT3, PTGS2, TFRC, APP, HMOX1, ALOX5, SLC7A11, CDK1, CDKN2A, EPAS1, BRCA1, NLRP3, IFNG, EIF2AK2, ACSL4, NEDD4, and CXCL8 (Figure 2).

Functional analysis of these hub genes revealed their close association with ferroptosis and inflammatory or immune regulation. Among them, SLC7A11, ACSL4, TFRC, and ALOX5 are established core regulators of ferroptosis, involved in glutathione synthesis, lipid metabolism, iron uptake, and lipid peroxidation, respectively [25,26,27,28]. Concurrently, TNF, IL6, IL1B, CXCL8, and NLRP3 are classical inflammatory factors playing crucial roles in the immune response and tissue damage triggered by *Candida* infection [29]. Furthermore, genes such as STAT3, PTGS2, and HMOX1 play dual roles in oxidative stress and immune modulation [30,31,32]. These findings suggest a potential synergistic interplay, where ferroptosis and the chronic inflammatory state in RVVC may mutually reinforce each other.

### 3.3. Ferroptosis Plays a Central Role in RVVC Through a Coordinated Multi-Pathway Network

To systematically elucidate the potential mechanistic roles of the ferroptosis-related genes in RVVC, we performed multi-layered functional and pathway enrichment analyses on the 194 identified key targets.

**Figure 3:**
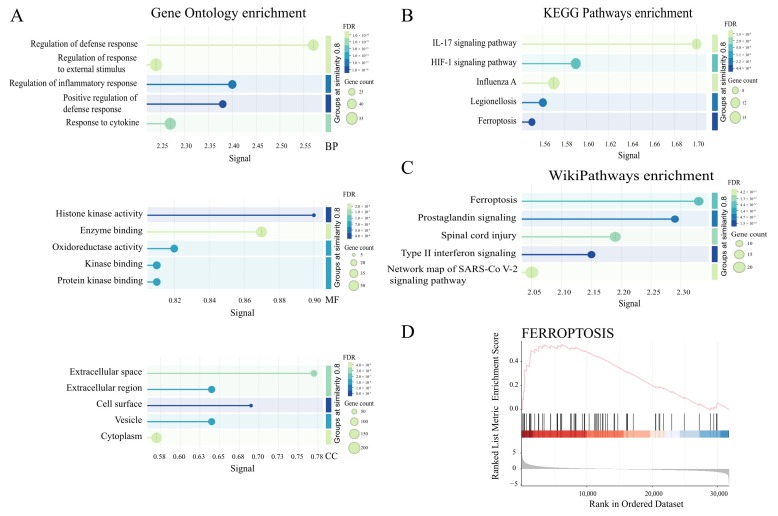
Functional and pathway enrichment analyses of RVVC-ferroptosis shared genes. (**A**) Gene Ontology (GO) enrichment analysis (three categories: Biological Process [BP], Molecular Function [MF], Cellular Component [CC]). Dot plots show significantly enriched GO terms: *x*-axis = enrichment signal, size of dots = number of genes annotated to the term, color gradient = FDR (higher value = more significant enrichment). (**B**) KEGG pathway enrichment analysis. Dot plot displays top enriched pathways: *x*-axis = enrichment signal, dot size = gene count, color = FDR. (**C**) WikiPathways enrichment analysis. Dot plot of significantly enriched pathways: *x*-axis = enrichment signal, dot size = gene count, color = FDR. (**D**) Gene Set Enrichment Analysis (GSEA) plot for the ferroptosis pathway in the RVVC DEGs (NES = 1.79, adj.*p* < 0.05). The curve reflects enrichment trend; vertical lines indicate positions of ferroptosis-related genes in the ranked DEG list.

First, GSEA of the RVVC DEGs revealed a significant enrichment trend for the ferroptosis pathway, providing preliminary evidence for its activated state within the overall gene expression profile of RVVC (NES = 1.79, adj.*p* < 0.05, Figure 3D). Subsequent GO functional annotation delineated the core biological characteristics of the shared genes. Biological Process: significantly enriched terms were concentrated in the regulation of defense response, response to external stimulus, and regulation of inflammatory response. Additionally, response to cytokine was highly enriched, indicating these genes primarily participate in RVVC pathogenesis by modulating host immune and inflammatory responses. Molecular Function: the most significantly enriched terms included histone kinase activity, enzyme binding, and oxidoreductase activity, with kinase binding and protein kinase binding also prominent. This suggests the genes predominantly function through enzymatic reactions and protein–protein interactions, closely related to lipid peroxidation in ferroptosis and the activation of inflammatory pathways. Cellular Component: the genes were primarily localized to the extracellular space, extracellular region, and cell surface, with enrichment also observed in vesicles and cytoplasm. This implicates their involvement in extracellular signaling and intracellular metabolic regulation, aligning with processes such as inflammatory cytokine secretion and iron ion transport (Figure 3A).

At the pathway level, KEGG enrichment analysis further pinpointed the core mechanisms. The top five significantly enriched pathways included the IL-17 signaling pathway, HIF-1 signaling pathway, ferroptosis pathway, Influenza A, and Legionellosis pathways. Among these, the IL-17 signaling pathway may be the core of the inflammatory response in RVVC [29], the HIF-1 pathway may links iron metabolism and inflammation under hypoxic conditions [33], and the direct enrichment of the ferroptosis pathway molecularly indicates the possibility that common targets participate in RVVC by regulating ferroptosis (Figure 3B).

To gain a more comprehensive view of the pathway network, we conducted a supplementary analysis using the WikiPathways database (https://www.wikipathways.org/, accessed on 2 November 2025) (Figure 3C). The results confirmed that the ferroptosis pathway was also the most significantly enriched WikiPathway, followed by the Prostaglandin Signaling pathway and Type II Interferon Signaling pathway, among others. This finding further solidifies that the shared genes collectively regulate the pathological process of RVVC through a synergistic “Ferroptosis-Inflammation-Immunity” multi-pathway network.

### 3.4. Analysis of Global Immune Infiltration Features in RVVC Patients and the Specific Immunoregulatory Pattern Associated with Ferroptosis-Related Genes

**Figure 4:**
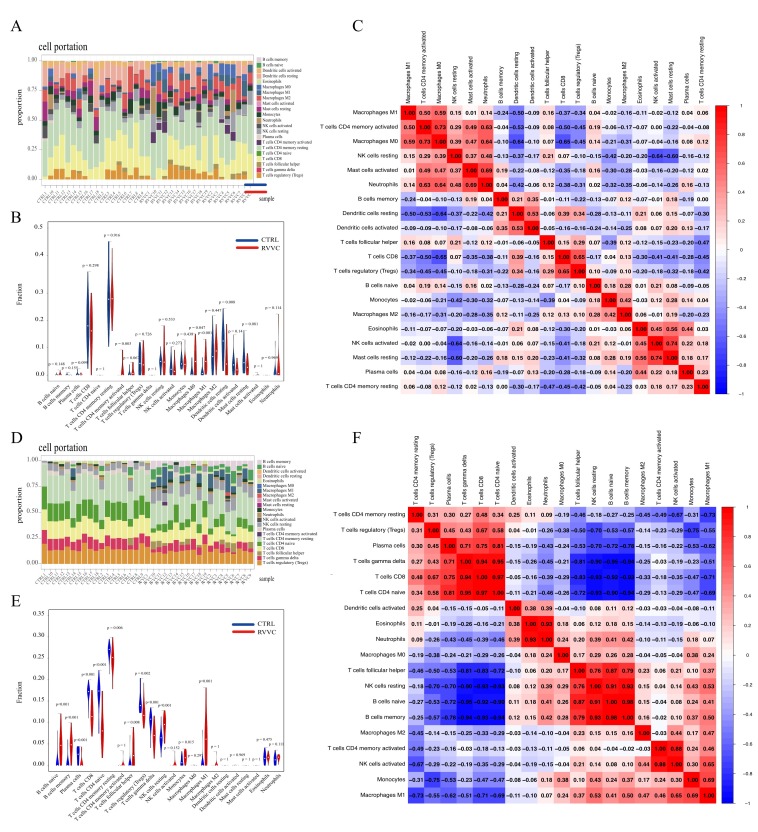
Immune infiltration features and ferroptosis-associated immune dysregulation in RVVC. (**A**) Distribution of immune cell proportions (by cell type) across samples (CTRL = healthy controls; RVVC = RVVC patients). (**B**) Violin plots of immune cell abundances in CTRL vs. RVVC groups (red = RVVC; blue = CTRL). (**C**) Correlation matrix of immune cells in RVVC: color intensity = correlation coefficient (red = positive; blue = negative); values = correlation coefficients (adj.*p* < 0.05). (**D**) Distribution of ferroptosis-associated immune cell proportions across samples. (**E**) Violin plots of ferroptosis-associated immune cell abundances in CTRL vs. RVVC groups (red = RVVC; blue = CTRL). (**F**) Correlation matrix of ferroptosis-associated immune cells in RVVC: color intensity = correlation coefficient (red = positive; blue = negative); values = correlation coefficients (adj.*p* < 0.05).

To elucidate the role of the immune microenvironment in the pathogenesis of RVVC, we systematically performed immune infiltration analysis on transcriptomic data from RVVC patients and healthy controls (CTRL). The results revealed a significant remodeling of the immune cell landscape in RVVC patients, characterized overall by a pro-inflammatory state. Specifically, the abundance of pro-inflammatory subsets, such as activated CD4 memory T cells and M1 macrophages, was significantly increased (Figure 4A,B). Correlation analysis further indicated that pro-inflammatory cells predominantly exhibited synergistic positive correlations, while associations among anti-inflammatory cells were markedly weakened, suggesting a global disruption of the immune network in RVVC (Figure 4C).

Building on this, to investigate the potential link between ferroptosis and the immune response, we focused on immune cell populations associated with ferroptosis-related genes (Figure 4D,E). This analysis uncovered a more specific pattern of immune dysregulation: core pro-inflammatory cells (e.g., M1 macrophages, follicular helper T cells, and monocytes) were significantly elevated, whereas key effector and regulatory populations, including plasma cells, CD8 T cells, naive CD4 T cells, resting CD4 memory T cells, regulatory T cells, and γδ T cells, were markedly downregulated. This pattern is closely related to sustained immune activation and effector function exhaustion caused by chronic inflammation. It reflects that persistent pathogen stimulation drives a pro-inflammatory response but fails to effectively activate pathogen-clearing adaptive immunity, even impairing its function.

Correlation network analysis further revealed that these core immune cells formed a highly synergistic “pro-inflammatory innate immune axis” that exhibited significant negative correlations with a functionally suppressed “adaptive immune axis” (Figure 4F). Specifically, M1 macrophages showed moderate positive correlations with monocytes (r = 0.69) and activated NK cells (r = 0.65), suggesting the activation of a pro-inflammatory cascade promoting monocyte differentiation towards the M1 phenotype. Follicular helper T cells showed strong positive correlations with naive B cells (r = 0.87) and memory B cells (r = 0.79), implying their potential role in mediating B cell activation biased towards a “non-protective humoral immune” response. Concurrently, M1 macrophages showed significant negative correlations with resting CD4 memory T cells (r = −0.69) and regulatory T cells (r = −0.55). Follicular helper T cells also exhibited strong negative correlations with γδ T cells (r = −0.81) and CD8 T cells (r = −0.83).

In conclusion, our immune infiltration analysis indicates that ferroptosis in RVVC is not merely a passive epiphenomenon but may serve as a key driver actively shaping the immune microenvironment.

### 3.5. Inhibition of Ferroptosis Alleviates Vaginal Histopathological Damage in a Murine Model of CVVC

The aforementioned bioinformatics analysis based on human RVVC transcriptomic data revealed significant enrichment of the ferroptosis pathway and its associated unique immune microenvironment, strongly suggesting a potential role for ferroptosis in RVVC pathogenesis. However, these findings are derived from predictive analyses, and the specific pathological function requires validation through in vivo experiments. Ferroptosis, an iron-dependent form of programmed cell death driven by lipid peroxidation, is characterized by core biochemical features: accumulation of lipid reactive oxygen species (Lipid ROS), iron ion (Fe^2+^) overload, and dysregulated expression of key regulatory proteins. This includes downregulation of glutathione peroxidase 4 (GPX4, a core enzyme inhibiting lipid peroxidation) and solute carrier family 7 member 11 (SLC7A11, a key transporter for glutathione synthesis), alongside upregulation of acyl-CoA synthetase long-chain family member 4 (ACSL4, an enzyme promoting generation of lipid peroxidation substrates). These changes are accompanied by the accumulation of malondialdehyde (MDA, a terminal product of lipid peroxidation) and 4-hydroxynonenal (4-HNE, a toxic lipid peroxidation product) [34,35].

Given that the core pathological features of RVVC include persistent inflammatory damage and fungal colonization, this section aimed to validate the involvement of ferroptosis in the RVVC pathological process at the tissue level. We established a murine model of CVVC and employed multi-dimensional detection techniques. Furthermore, we explored the therapeutic potential of the ferroptosis inhibitor Fer-1. These investigations were conducted to provide a novel perspective on the pathogenesis of RVVC.

#### Detection of Ferroptosis Levels in Vaginal Tissue Cells from CVVC Mice

Lipid peroxidation.

**Figure 5:**
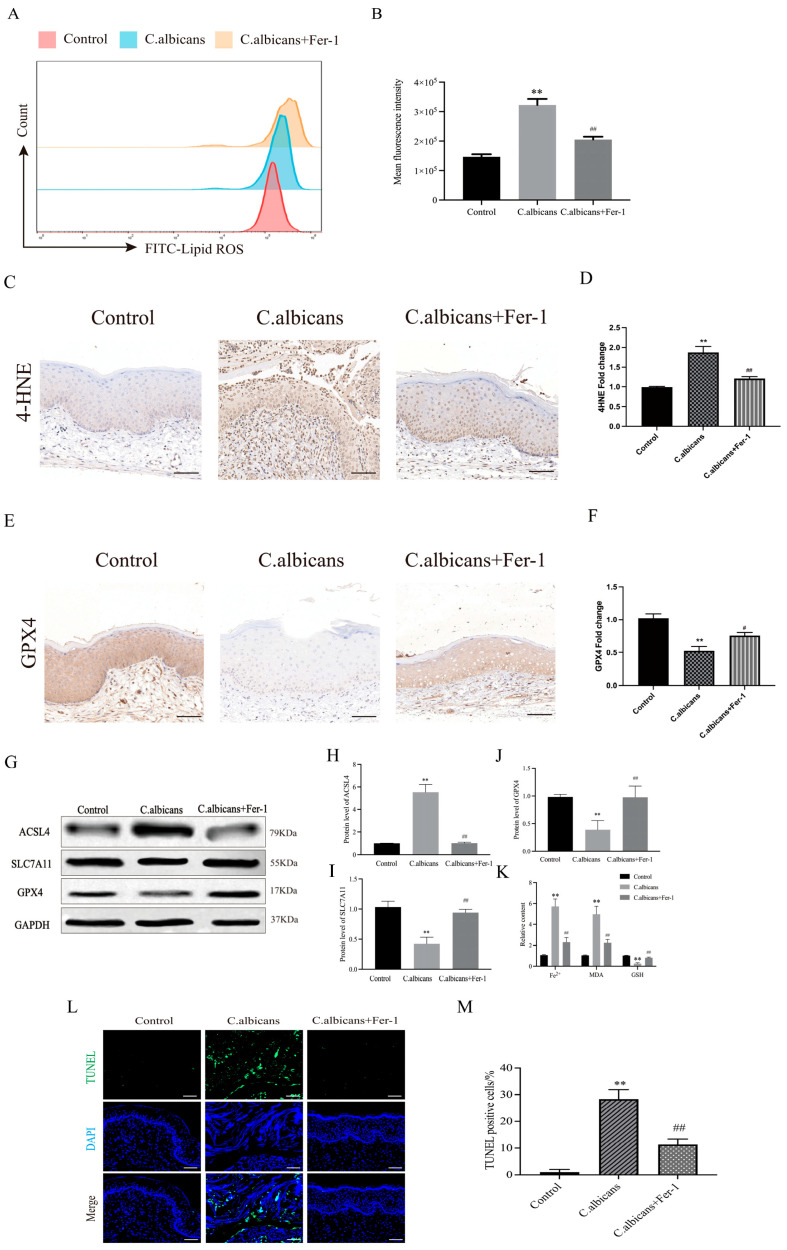
Detection of ferroptosis levels in vaginal tissue cells from CVVC mice. (**A**) Flow cytometry showing the intensity of BODIPY 581/591 C11 fluorescence in vaginal tissue cells across groups: Control (control group), *C. albicans* (*C. albicans*-infected group), *C. albicans*+Fer-1 (*C. albicans*-infected group + Fer-1). (**B**) Quantitative analysis of the mean fluorescence intensity (MFI) of Lipid ROS from flow cytometry data. (**C**) IHC staining of 4-HNE in vaginal tissue sections (Brown deposits indicate positive staining. bar = 50 μm, ×200). (**D**) Quantitative analysis of the 4-HNE positive area from IHC images. (**E**) IHC staining of GPX4 in vaginal tissue sections (Brown deposits indicate positive staining. bar = 50 μm, ×200). (**F**) Quantitative analysis of the GPX4 positive area. (**G**) Western blot bands showing protein levels of GPX4, SLC7A11, ACSL4, and GAPDH. (**H**–**J**) Quantitative analysis of protein expression (normalized to GAPDH). (**K**) Bar plots showing relative levels of (left to right) Fe^2+^, MDA, and GSH in vaginal tissues. (**L**) TUNEL (green, labels DNA-fragmented cells) and DAPI (blue, nuclear stain) co-staining of vaginal tissue sections (bar = 50 μm, ×200; Merge panels show colocalization of TUNEL and DAPI signals. (**M**) Quantitative analysis of TUNEL-positive cell percentage. Data are presented as mean ± SD (n = 5 mice per group); groups: Control (control group), *C. albicans* (*C. albicans*-infected group), *C. albicans*+Fer-1 (*C. albicans*-infected group + Fer-1); * *p* < 0.05 vs. Control; ** *p* < 0.01 vs. Control; # *p* < 0.05 vs. *C. albicans* group; ## *p* < 0.01 vs. *C. albicans* group.

Lipid ROS serve as a direct driver of ferroptosis, and sustained lipid ROS accumulation disrupts membrane integrity and promotes cell death [36]. Flow cytometry revealed that the proportion of lipid ROS-positive vaginal tissue cells was significantly increased in the *C. albicans*-infected group compared with controls, whereas Fer-1 markedly reduced lipid ROS levels (Figure 5A,B). Consistently, IHC staining showed enhanced 4-HNE deposition in vaginal mucosa/submucosa after infection, which was attenuated by Fer-1 (Figure 5C,D), supporting infection-driven lipid peroxidation in vivo. These results indicate that RVVC is associated with an enhanced lipid peroxidation burden in vivo.

Iron metabolism and antioxidant capacity.

Because ferroptosis is iron-dependent and tightly coupled to antioxidant depletion, we quantified Fe^2+^, MDA, and GSH levels in vaginal tissues. Infection significantly increased Fe^2+^ and MDA, while decreasing GSH, indicating iron accumulation and impaired redox buffering; Fer-1 reversed these changes (Figure 5K). Notably, although Fe^2+^/MDA/GSH are displayed together for integrated visualization of the iron–lipid peroxidation–antioxidant axis, they reflect two mechanistically linked components: iron overload (Fe^2+^) and oxidative lipid injury (MDA) accompanied by antioxidant exhaustion (GSH).

Ferroptosis regulatory proteins.

At the regulatory level, IHC indicated reduced GPX4 expression in infected vaginal tissues and restoration after Fer-1 treatment (Figure 5E,F). Furthermore, Western blot analysis demonstrated upregulation of the pro-ferroptotic protein ACSL4 and downregulation of anti-ferroptotic proteins GPX4 and SLC7A11 following infection, all of which were significantly reversed by Fer-1 (Figure 5G–J).

Cell death assays

Finally, TUNEL staining revealed increased TUNEL-positive cells in infected vaginal tissues, predominantly localized to areas of mucosal epithelial injury and shed vaginal cells, while Fer-1 significantly reduced TUNEL positivity (Figure 5L,M). Although TUNEL does not distinguish ferroptosis from other cell death programs, combining these findings with lipid peroxidation, iron/antioxidant imbalance, and ferroptosis marker changes supports the presence of ferroptosis-associated tissue injury in CVVC [37].

### 3.6. Inhibition of Ferroptosis Enhances Host Fungal Clearance and Reduces Fungal Burden

A key clinical challenge in RVVC is persistent fungal colonization and recurrent infection. This section aimed to test the hypothesis that “inhibition of ferroptosis can promote fungal clearance” by measuring the fungal burden in vaginal lavage fluid following Fer-1 intervention, thereby providing evidence for the clinical value of targeting ferroptosis.

**Figure 6:**
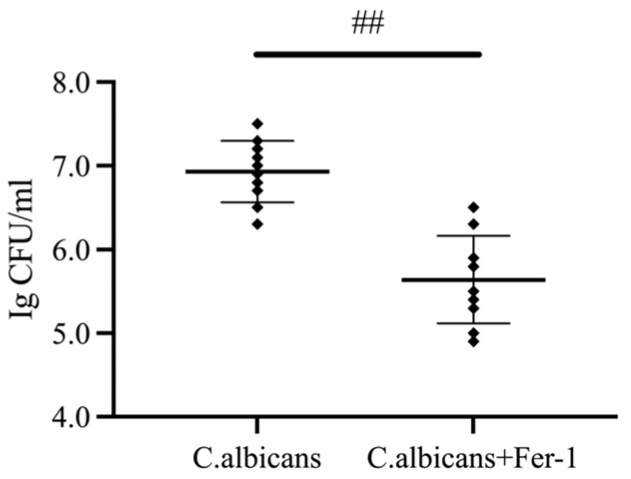
Fer-1 reduces *C. albicans* burden in vaginal tissue of CVVC mice. Dot plot showing fungal burden (expressed as log CFU/mL; n = 10 mice per group) in vaginal lavage fluid of *C. albicans* vs. *C. albicans*+Fer-1 groups. Horizontal lines represent mean ± SD; ## *p* < 0.01 vs. *C. albicans* group.

As shown in Figure 6, compared to the *C. albicans*-infected group, the number of *C. albicans* CFU in the vaginal lavage fluid was significantly reduced in the Fer-1-treated group (*p* < 0.05). This result suggests that ferroptosis activation may contribute to a microenvironment that is permissive for *C. albicans* persistence.

### 3.7. Macrophages Are Key Target Cells Undergoing Ferroptosis

Notably, the abundance of M1 macrophages was significantly upregulated both in the previous global immune infiltration analysis and within the immune population defined by ferroptosis-related genes, highlighting their central role in remodeling the RVVC immune microenvironment. This overlap strongly suggests that the aberrant activation of M1 macrophages is not merely part of a general inflammatory response but may be functionally coupled with the specific cellular metabolic death program of ferroptosis.

As the core cellular component of the local anti-fungal immunity in the vagina, macrophages play a crucial role in maintaining vaginal microenvironment homeostasis by phagocytosing and killing fungi and secreting anti-inflammatory factors to regulate the inflammatory response [29,38,39]. We hypothesize that under *C. albicans* stimulation, over-activated M1 macrophages may experience severe oxidative stress and lipid peroxidation, making them prone to ferroptosis. This process could have dual pathological significance: firstly, the aberrant death of macrophages weakens their ability to clear pathogens; secondly, the pro-inflammatory signals released during ferroptosis further sustain local inflammation and impair adaptive immune responses. Therefore, macrophage ferroptosis may be a key link connecting chronic infection, persistent inflammation, and immune failure. Investigating whether macrophages undergo ferroptosis will provide mechanistic insights into immune failure in RVVC.

**Figure 7:**
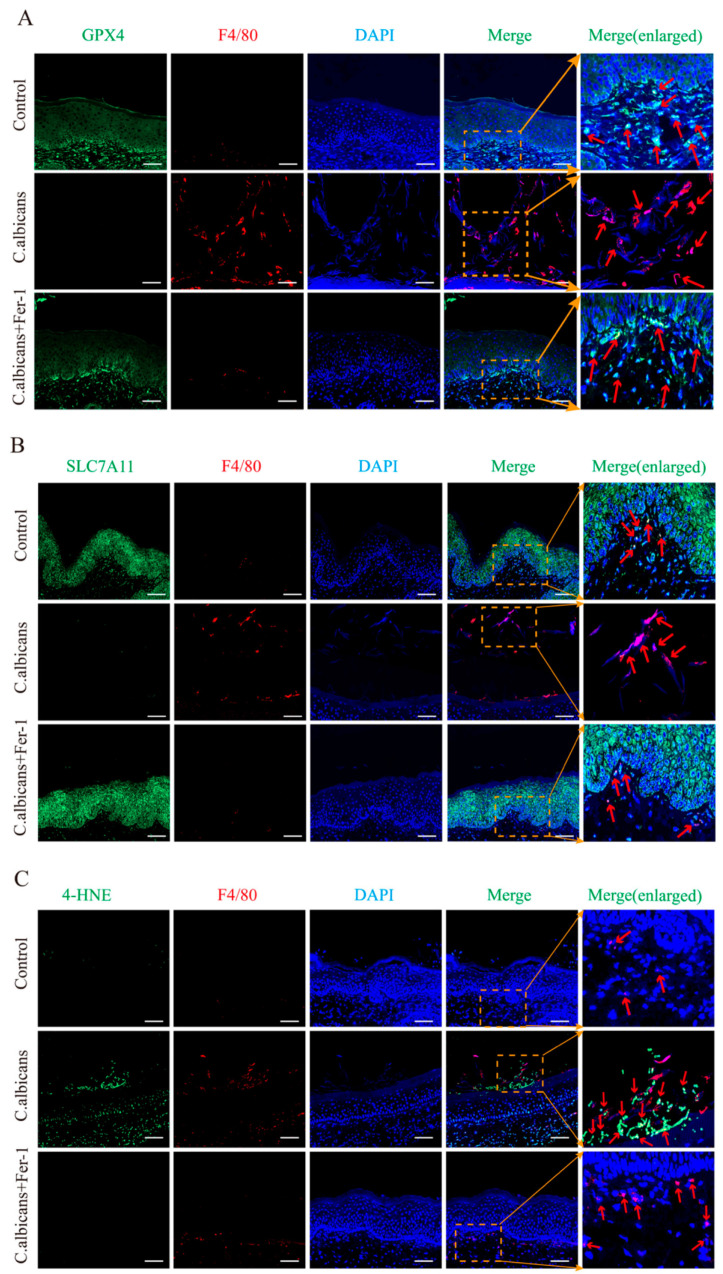
*C. albicans* infection induces ferroptosis in vaginal macrophages, which is reversed by Fer-1 treatment. (**A**–**C**) Representative IF co-staining images of vaginal tissue sections from Control, *C. albicans*, and *C. albicans*+Fer-1 mice (groups: Control (control group), *C. albicans* (*C. albicans*-infected group), *C. albicans*+Fer-1 (*C. albicans*-infected group + Fer-1)). Sections were stained for the macrophage marker F4/80 (red) and key ferroptosis-related proteins (green): (**A**) 4-HNE; (**B**) GPX4; (**C**) SLC7A11. Nuclei are counterstained with DAPI (blue). bar = 50 μm, ×200; Merged and enlarged views of the boxed regions are shown (Red arrows indicate the locations of macrophages). (n = 5 mice per group).

Co-staining using the macrophage-specific marker F4/80 and ferroptosis indicators (4-HNE, GPX4, SLC7A11) revealed that in the control group, F4/80^+^ macrophages exhibited very weak 4-HNE fluorescence signal, while GPX4 and SLC7A11 fluorescence signals were strong and uniform. In the *C. albicans* infection group, the fluorescence intensity of 4-HNE within F4/80^+^ macrophages was significantly enhanced (*p* < 0.05), while the fluorescence intensities of GPX4 and SLC7A11 were significantly weakened (*p* < 0.05). Following Fer-1 intervention, the 4-HNE fluorescence signal in F4/80^+^ macrophages was significantly reduced (*p* < 0.05), and the fluorescence signals of GPX4 and SLC7A11 were restored to levels close to normal (*p* > 0.05). These results indicate that *C. albicans* infection specifically induces ferroptosis in vaginal macrophages, and Fer-1 can effectively reverse this process (Figure 7A–C).

**Figure 8:**
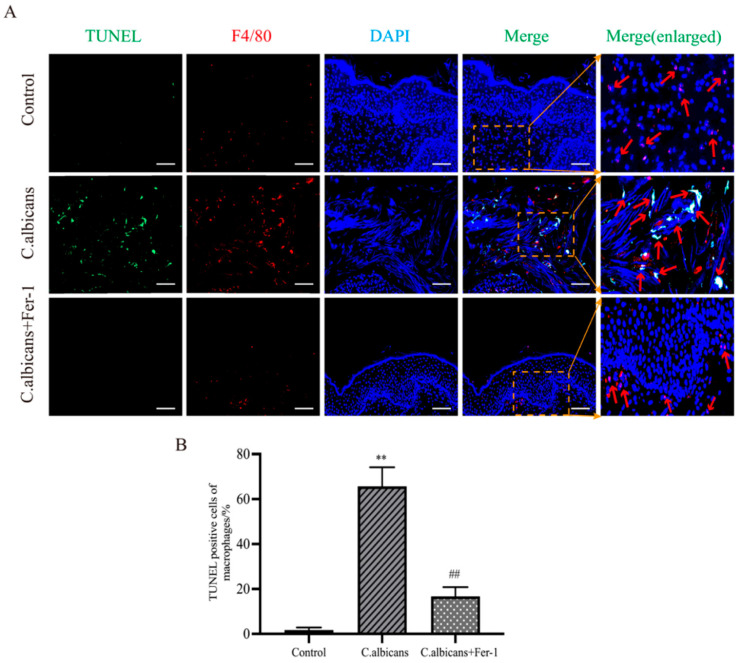
Quantification of macrophage death in vaginal tissues of CVVC mice and its attenuation by Fer-1. (**A**) IF co-staining of vaginal tissue sections (groups: Control (control group), *C. albicans* (*C. albicans*-infected group), *C. albicans*+Fer-1 (*C. albicans*-infected group + Fer-1)): TUNEL (green, DNA-fragmented cells) + F4/80 (red, macrophage marker) + DAPI (blue, nuclei). Merge panels (and enlarged insets, red arrows = F4/80^+^TUNEL^+^/-cells) show macrophage signals. bar = 50 μm, ×200; n = 5 mice per group. (**B**) Quantitative analysis of F4/80^+^TUNEL^+^ double-positive cell percentage. Data are mean ± SD; * *p* < 0.05 vs. Control; ** *p* < 0.01 vs. Control; # *p* < 0.05 vs. *C. albicans* group; ## *p* < 0.01 vs. *C. albicans* group.

To further quantify the proportion of ferroptosis-associated cell death in macrophages, co-staining of TUNEL and F4/80 was performed. The results showed a low proportion of F4/80^+^TUNEL^+^ double-positive cells in the normal control group. In contrast, the proportion of double-positive cells was significantly increased in the *C. albicans* infection group (*p* < 0.05). Following Fer-1 intervention, this proportion decreased (*p* < 0.05). These results directly confirm that macrophage death constitutes a significant component of the overall cell death in vaginal tissue during chronic inflammation. Moreover, inhibiting ferroptosis significantly reduces macrophage death, thereby helping to preserve their anti-fungal function (Figure 8A,B).

### 3.8. Inhibition of Ferroptosis Directly Protects Macrophage Function and Enhances Their Antifungal Activity

BMDMs serve as a classical in vitro model for studying macrophage function, with biological characteristics highly consistent with tissue-resident macrophages in vivo [40]. Given that in vivo experiments have confirmed ferroptosis in vaginal macrophages, this section utilized an in vitro “*C. albicans*-BMDMs” infection model to explore, without the interference of the complex in vivo microenvironment (e.g., hormones, other immune cells), whether *C. albicans* directly induces ferroptosis in macrophages and whether ferroptosis affects macrophage viability and antifungal function.

As shown in Supplement Figure S2A,B, the purity of the isolated BMDMs was validated using flow cytometry and immunofluorescence. Flow cytometric analysis indicated that over 98% of the cells were positive for the macrophage-specific surface antigens F4/80 and CD11b. Furthermore, fluorescence microscopy revealed green fluorescence from FITC-labeled F4/80 on the BMDM surface, confirming the high purity of the isolated BMDMs suitable for subsequent experiments.

Cell viability is a prerequisite for macrophages to perform phagocytosis and killing functions, and the lactate dehydrogenase (LDH) release rate is a common indicator reflecting cell membrane integrity (i.e., the extent of cell death) [41]. The fungicidal capacity of macrophages against *C. albicans* directly reflects their antifungal function and is a core metric for evaluating immune efficacy. This section utilized LDH release assays and fungal killing assays to validate the impact of ferroptosis on the antifungal function of BMDMs from the two dimensions of “cell survival” and “functional performance.”

The LDH release assay results showed that compared to the control group, the LDH release rate was significantly increased in BMDMs from the *C. albicans* infection group (*p* < 0.05), indicating that infection caused BMDM cell membrane damage and cell death. In contrast, the LDH release rate was significantly reduced in the Fer-1 treatment group (*p* < 0.05), suggesting that Fer-1 could reduce BMDMs death and maintain cell viability by inhibiting ferroptosis.

**Figure 9:**
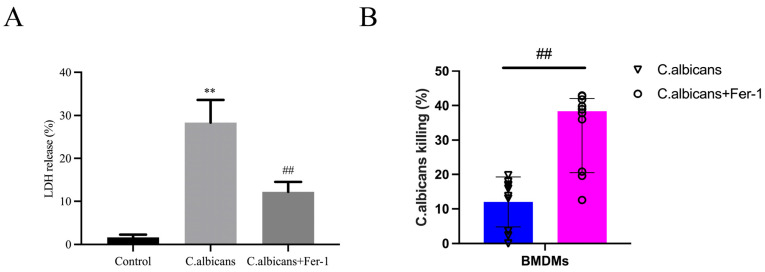
Fer-1 preserves BMDMs viability and enhances their *C. albicans* killing capacity. (**A**) LDH release rate in BMDMs across groups. Data are mean ± SD; * *p* < 0.05 vs. Control; ** *p* < 0.01 vs. Control; # *p* < 0.05 vs. *C. albicans* group; ## *p* < 0.01 vs. *C. albicans* group. (**B**) *C. albicans* killing rate of BMDMs in *C. albicans* vs. *C. albicans*+Fer-1 groups. Data are individual values (dots) with group distributions (n = 3 independent experiments); ## *p* < 0.01 vs. *C. albicans* group; groups: Control (control group), *C. albicans* (*C. albicans*-infected BMDMs group), *C. albicans*+Fer-1 (*C. albicans*-infected BMDMs group + Fer-1). Data are median (interquartile range).

The fungal killing assay results are shown in Figure 9A,B. Compared to the Fer-1 treatment group, the killing rate was significantly decreased in the *C. albicans* infection group (*p* < 0.05), indicating that infection-induced ferroptosis likely impaired the killing function of BMDMs. Combining this with the LDH results, it can be inferred that the restored cell viability resulting from the inhibition of BMDMs ferroptosis forms an important foundation for the enhancement of their antifungal killing capacity. This finding clarifies that ferroptosis is a key regulatory target for macrophage antifungal function and provides functional evidence for subsequent mechanistic studies.

**Figure 10:**
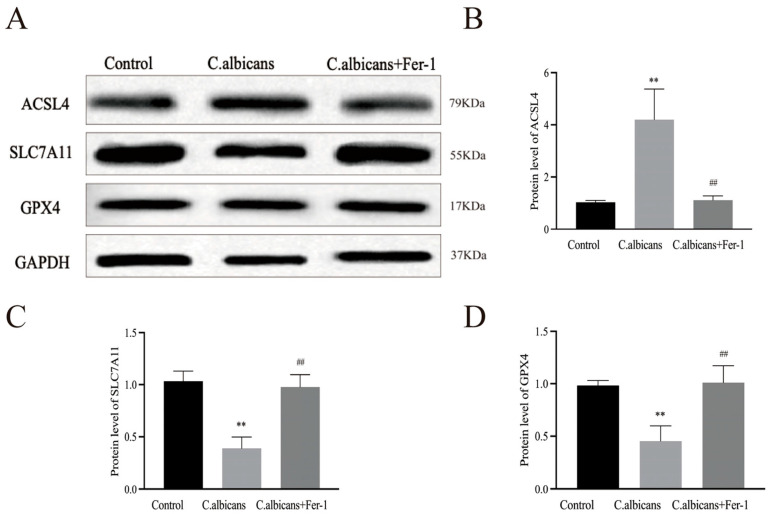
Fer-1 modulates ferroptosis core regulatory proteins in *C. albicans*-infected BMDMs. (**A**) Western blot bands showing protein levels of GPX4, SLC7A11, ACSL4, and GAPDH in BMDMs (groups: Control (control group), *C. albicans* (*C. albicans*-infected BMDMs group), *C. albicans*+Fer-1 (*C. albicans*-infected BMDMs group + Fer-1)). (**B**–**D**) Quantitative analysis of protein expression (normalized to GAPDH). Data are mean ± SD (n = 3 independent experiments); * *p* < 0.05 vs. Control; ** *p* < 0.01 vs. Control; # *p* < 0.05 vs. *C. albicans* group; ## *p* < 0.01 vs. *C. albicans* group.

Western blot analysis further revealed that compared to the control group, the protein expression of ACSL4 was significantly upregulated in BMDMs from the *C. albicans* infection group (*p* < 0.05), while the expression of GPX4 and SLC7A11 was significantly downregulated (*p* < 0.05 for both). Following Fer-1 intervention, the expression of ACSL4 was decreased (*p* < 0.05), whereas the expression of GPX4 and SLC7A11 was increased (*p* < 0.05) (Figure 10A–D).

**Figure 11:**
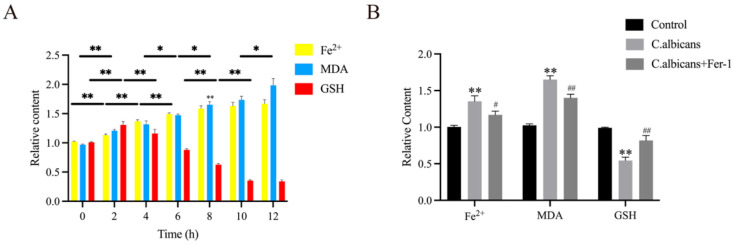
*C. albicans* infection disrupts iron/antioxidant homeostasis in BMDMs, reversed by Fer-1. (**A**) Time-course changes in relative content of Fe^2+^ (yellow), MDA (blue), and GSH (red) in *C. albicans*-infected BMDMs. Data are mean ± SD; * *p* < 0.05, ** *p* < 0.01 vs. baseline (0 h). (**B**) Quantitative analysis of Fe^2+^, MDA, and GSH levels in BMDMs (groups: Control (control group), *C. albicans* (*C. albicans*-infected BMDMs group), *C. albicans*+Fer-1 (*C. albicans*-infected BMDMs group + Fer-1)). Data are mean ± SD (n = 3 independent experiments); * *p* < 0.05 vs. Control; ** *p* < 0.01 vs. Control; # *p* < 0.05 vs. *C. albicans* group; ## *p* < 0.01 vs. *C. albicans* group.

Furthermore, as shown in Figure 11A, following *C. albicans* infection of BMDMs, the levels of Fe^2+^ and MDA showed a general increasing trend over time. The level of GSH exhibited a similar trend to GPX4 protein expression, initially increasing during the early stages of infection but subsequently declining in the later stages. It is speculated that GSH, as a substrate for GPX4, may confer a degree of resistance against ferroptosis in the early phase by supporting GPX4 activity. In contrast, during the late phase, with the accumulating burden of iron and lipid peroxides, GSH consumption likely increases.

Concurrently, as shown in Figure 11B, compared to *C. albicans*-infected BMDMs, Fer-1 treatment mitigated the decrease in GSH levels and the increase in Fe^2+^ and MDA levels (*p* < 0.05). These results further confirm that *C. albicans* infection disrupts the iron metabolism balance and the homeostasis of the antioxidant system in BMDMs. Conversely, Fer-1 can block the lipid peroxidation cascade by reducing Fe^2+^ accumulation and restoring GSH levels.

**Figure 12:**
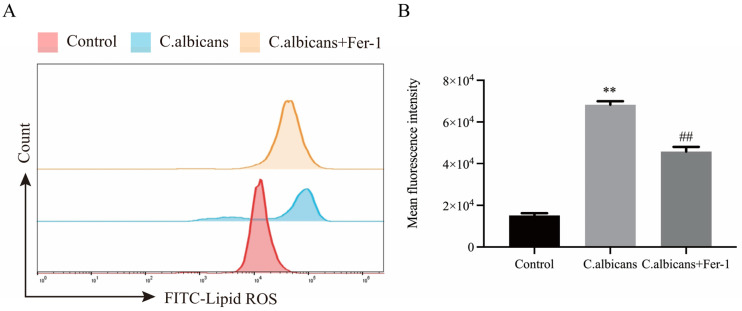
Fer-1 inhibits *C. albicans*-induced lipid ROS accumulation in BMDMs. (**A**) Flow cytometry of FITC-labeled lipid ROS levels in BMDMs across groups using BODIPY 581/591 C11 probe. (**B**) Quantitative analysis of lipid ROS MFI from flow cytometry. groups: Control (control group), *C. albicans* (*C. albicans*-infected BMDMs group), *C. albicans*+Fer-1 (*C. albicans*-infected BMDMs group + Fer-1); Data are mean ± SD (n = 3 independent experiments); * *p* < 0.05, ** *p* < 0.01 vs. Control; ## *p* < 0.01 vs. *C. albicans* group.

Concurrently, as shown in Figure 12A,B and Figure S3A,B, we assessed the levels of lipid ROS in *C. albicans*-infected BMDMs under the influence of Fer-1 using flow cytometry and IF. The results demonstrated that Fer-1 significantly inhibited the *C. albicans*-induced production of lipid ROS in BMDMs (*p* < 0.05).

**Figure 13:**
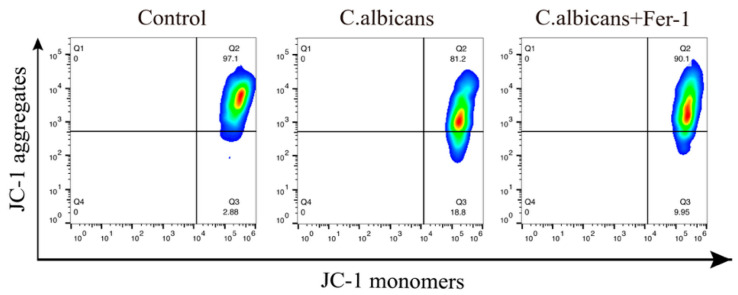
Fer-1 restores MMP in *C. albicans*-infected BMDMs. Representative flow cytometry density plots of JC-1 staining in BMDMs. groups: Control (control group), *C. albicans* (*C. albicans*-infected BMDMs group), *C. albicans*+Fer-1 (*C. albicans*-infected BMDMs group + Fer-1); the shift from the upper right (high red/green ratio) to the lower right (low red/green ratio) quadrant in the *C. albicans* group indicates mitochondrial depolarization, which is partially restored by Fer-1 treatment (Representative images from 3 independent experiments).

Lipid ROS, serving as direct effector molecules of ferroptosis, accumulate and cause direct damage to mitochondria, the key target organelles in ferroptosis. MMP is a critical indicator for assessing mitochondrial function [42]. Therefore, this study utilized the JC-1 probe to evaluate MMP levels in *C. albicans*-infected BMDMs via IF and flow cytometry. As shown in Figure 13 and Figure S4, infection with *C. albicans* led to a significant decrease in red fluorescence intensity (indicative of JC-1 aggregates) and a significant increase in green fluorescence intensity (indicative of JC-1 monomers) in BMDMs, representing a reduction in MMP. Fer-1 treatment was able to reverse these alterations.

**Figure 14:**
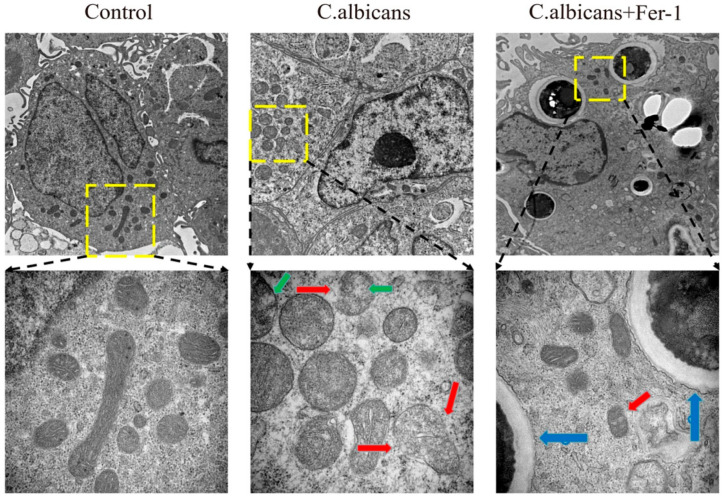
*C. albicans* infection induces ferroptosis-characteristic mitochondrial damage in BMDMs, mitigated by Fer-1. TEM images of BMDM mitochondria (yellow dashed boxes indicate magnified regions): Control (control group): Mitochondria show intact membranes and dense cristae. *C. albicans* (*C. albicans*-infected BMDMs group): Mitochondria exhibit ferroptosis-specific morphology: membrane rupture (red arrows) and reduced/disappeared cristae (green arrows). *C. albicans*+Fer-1 (*C. albicans*-infected BMDMs group + Fer-1): Mitochondrial damage is alleviated (blue arrows = *C. albicans*), (bar 2/200 μm, ×4000/50,000, Representative TEM images from 3 independent experiments (independent cell preparations).

Alterations in mitochondrial morphology represent a specific hallmark distinguishing ferroptosis from apoptosis and necrosis. During ferroptosis, mitochondria typically exhibit swelling, cristae rupture or disappearance, and outer membrane rupture, whereas apoptosis is often characterized by cristae condensation and apoptotic body formation [43]. As shown in Figure 14, this study employed TEM to examine the ultrastructure of mitochondria within BMDMs. Compared to the control group, mitochondria in BMDMs exposed to *C. albicans* exhibited membrane rupture and reduced or disappeared cristae, which are typical cellular morphological features of ferroptosis. Treatment with Fer-1 reduced the extent of this mitochondrial damage. Collectively, these data provide further in vitro experimental evidence demonstrating that *C. albicans* infection induces ferroptosis in macrophages.

### 3.9. Ferroptosis Exacerbates Vaginal Tissue Damage and Inflammatory Responses in CVVC Mice

Beyond fungal colonization, the core pathological features of RVVC include destruction of the vaginal mucosal structure and local immune-inflammatory imbalance, both of which contribute to the persistent nature of the disease [44]. Therefore, this section systematically investigated the regulatory role of ferroptosis on the homeostasis of the local vaginal microenvironment in CVVC mice, aiming to further elucidate the role of ferroptosis within the pathological cascade of RVVC.

Vaginal mucosal epithelial integrity serves as a physical barrier against *C. albicans* invasion. Epithelial structural damage and inflammatory cell infiltration are hallmark manifestations of RVVC tissue injury. LDH, an intracellular enzyme, is released upon cell membrane damage, and its level in lavage fluid serves as an objective indicator for assessing tissue cell injury. This study evaluated the association between ferroptosis and vaginal tissue damage in RVVC by observing histomorphological changes via H&E staining and quantifying cellular damage through LDH detection.

**Figure 15:**
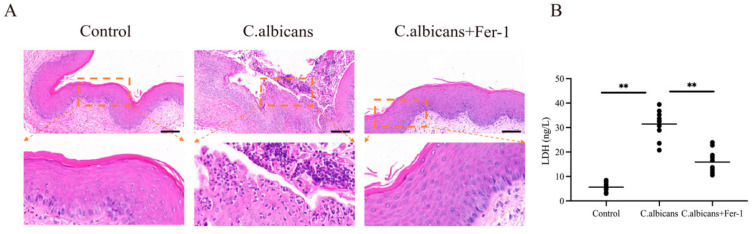
Fer-1 alleviates vaginal tissue damage in CVVC mice. (**A**) H&E-stained sections of vaginal tissue from Control (control group), *C. albicans* (*C. albicans*-infected group), *C. albicans*+Fer-1 (*C. albicans*-infected group + Fer-1) mice. Dashed boxes indicate regions shown at higher magnification. The Control group shows intact mucosal epithelium with a continuous superficial keratinized layer and no significant inflammatory infiltration. The *C. albicans* group exhibits loss of the keratinized layer, disorganized squamous epithelium, and prominent inflammatory cell aggregation. Fer-1 treatment restored the keratinized/squamous epithelial structure and reduced inflammatory infiltration. (bar = 50 μm, ×200; n = 5 mice per group). (**B**) Quantitative analysis of LDH activity in vaginal lavage fluid. n = 10 mice per group. Data are mean ± SD; * *p* < 0.05 vs. Control; ** *p* < 0.01 vs. Control; # *p* < 0.05 vs. *C. albicans* group; ## *p* < 0.01 vs. *C. albicans* group.

As shown in Figure 15A, vaginal tissues from the control group exhibited clear stratification, intact mucosal epithelium, a continuous superficial keratinized layer, and no significant inflammatory cell infiltration in the submucosa. In contrast, tissues from the *C. albicans*-infected group showed loss of the superficial keratinized layer, disorganized hyperplasia of the squamous epithelium, and substantial aggregation of inflammatory cells within the mucosal tissue. Fer-1 treatment alleviated inflammatory cell infiltration and restored the structure of the superficial keratinized layer and squamous epithelium.

The LDH activity in vaginal lavage fluid is shown in Figure 15B. Compared to the control group, LDH activity was significantly increased in the *C. albicans*-infected group (*p* < 0.01), indicating that infection-induced ferroptosis led to the loss of cell membrane integrity and consequent LDH release. Following Fer-1 intervention, LDH activity showed a significant decrease (*p* < 0.05). These findings further support the involvement of ferroptosis activation in cellular damage within vaginal tissue during chronic inflammation, and suggest that inhibiting ferroptosis can mitigate tissue injury, at least in part, by preserving cell membrane integrity.

### 3.10. Effect of Ferroptosis on Cytokine Secretion in Vaginal Tissue of CVVC Mice

Inflammatory cytokines are central molecules regulating the local immune response in RVVC. The dynamic balance between pro-inflammatory and anti-inflammatory factors directly influences disease outcome—excessive inflammation can enhance antifungal effects but also aggravate tissue damage, while insufficient inflammation suppression may hinder fungal clearance [44,45]. Based on the finding that Fer-1 reduces inflammatory cell infiltration, this study further employed the AAM-INF-1 inflammatory cytokine array [46] to screen for key cytokines regulated by ferroptosis, aiming to clarify the impact of ferroptosis on the inflammatory microenvironment in vaginal tissue.

Comparison of Inflammatory Cytokine Profiles in Vaginal Tissue between Control and *C. albicans*-Infected Mice.

**Figure 16:**
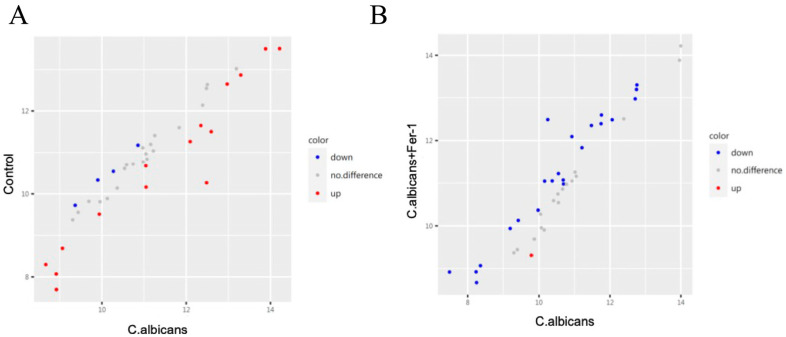
Effect of ferroptosis on cytokine secretion in vaginal tissue of CVVC mice. (**A**) Between Control and *C. albicans*. (**B**) Between *C. albicans* and *C. albicans*+Fer-1; scatter plot comparing cytokine expression levels. Dots are colored by expression trend: blue = downregulated, gray = no difference, red = upregulated in the infected group. groups: Control (control group), *C. albicans* (*C. albicans*-infected group), *C. albicans*+Fer-1 (*C. albicans*-infected group + Fer-1).

As shown in Figure 16A and Supplement Table S2, a comparison between the control and *C. albicans*-infected groups revealed 19 significant DEPs in mouse vaginal tissue. Among these, 4 proteins were downregulated and 15 proteins were upregulated in the infected group. The upregulated proteins included various pro-inflammatory and anti-inflammatory cytokines, indicating a state of high inflammatory activity in the vaginal tissue of CVVC mice.

Comparison of Inflammatory Cytokine Profiles in Vaginal Tissue between *C. albicans*-Infected Mice and Fer-1-Treated *C. albicans*-Infected Mice

As shown in Figure 16B and Supplement Table S3, a comparison between the *C. albicans*-infected group and the Fer-1-treated *C. albicans*-infected group revealed 23 DEPs in mice vaginal tissue. Among these, 22 proteins were downregulated and 1 protein was upregulated in the Fer-1-treated group. Notably, the downregulated proteins included I-TAC, TIMP-1, TIMP-2, S100A8, MCP-1, IFN-γ, TNF-α, IL-2, RANTES, IL-1β, G-CSF, IL-17, and IL-4, all of which were cytokines that had shown significant elevation in the *C. albicans*-infected group compared to the control group. These results suggest that Fer-1 treatment can effectively counteract the upregulation of numerous inflammatory cytokines observed in the vaginal tissue of *C. albicans*-infected mice.

### 3.11. Effects of Inhibiting Ferroptosis on the Secretion of TNF-α, IL-1β, IL-17, IFN-γ, IL-4, and IL-2 in Vaginal Lavage Fluid of CVVC Mice and Its Immunoregulatory Role

Transcriptomic data indicated a significant increase in the abundance of pro-inflammatory subsets, such as activated CD4 memory T cells and M1 macrophages, in RVVC patients. Among these, the balance between CD4^+^ T cell subsets—Th1 (secreting TNF-α, IFN-γ, IL-2) and Th2 (secreting IL-4)—is particularly crucial. Existing research has confirmed that an imbalance in vaginal local Th1/Th2 immunity (skewed towards a Th2-dominant response) in VVC patients weakens anti-fungal immunity, leading to persistent fungal colonization [47,48]. Integrating the results from the cytokine array, hub gene identification, and pathway enrichment analyses, this study used ELISA to measure the levels of key cytokines (TNF-α, IL-1β, IL-17, IFN-γ, IL-4, IL-2) in vaginal lavage fluid. The Th1/Th2 cytokine ratios were calculated to elucidate the regulatory mechanism of ferroptosis on immune balance.

**Figure 17:**
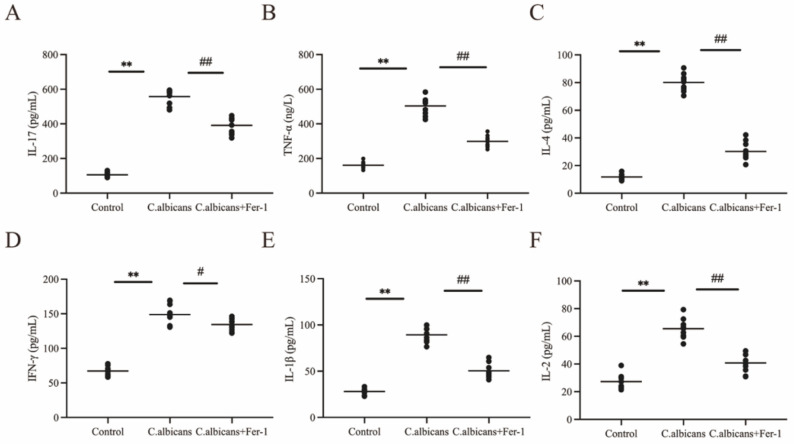
Fer-1 normalizes key cytokine levels. (**A**–**F**) Levels of key cytokines in vaginal lavage fluid were quantified by ELISA. Mice were treated as follows: Control (control group), *C. albicans* (*C. albicans*-infected group), *C. albicans*+Fer-1 (*C. albicans*-infected group +Fer-1). (**A**) IL-17; (**B**) TNF-α; (**C**) IL-4; (**D**) IFN-γ; (**E**) IL-1β; (**F**) IL-2. Data are presented as mean ± SD (n = 10 mice per group). * *p* < 0.05 vs. Control; ** *p* < 0.01 vs. Control; # *p* < 0.05 vs. *C. albicans* group; ## *p* < 0.01 vs. *C. albicans* group.

ELISA results are shown in Figure 17, compared to the control group, the levels of TNF-α (a Th1 pro-inflammatory cytokine), IL-1β (a pro-inflammatory cytokine), IL-17 (a Th17 pro-inflammatory cytokine), IFN-γ (a core Th1 cytokine), IL-4 (a core Th2 cytokine), and IL-2 (a Th1 proliferation factor) in the vaginal lavage fluid of the *C. albicans*-infected group were all significantly elevated (*p* < 0.01). This indicates that infection-induced ferroptosis not only activates a pro-inflammatory response but also causes a comprehensive upregulation of both Th1 and Th2 cytokines. Following Fer-1 intervention, the levels of all six cytokines decreased significantly (*p* < 0.05), confirming that ferroptosis acts as an upstream regulatory node for these key inflammatory factors.

Analysis of Th1/Th2 cytokine ratios is shown in Supplement Table S4: In the *C. albicans*-infected group, although both Th1 and Th2 cytokines were upregulated, the increase in the Th2 cytokine IL-4 was more pronounced. This led to a significant decrease in the Th1/Th2 ratios (*p* < 0.01), suggesting that infection-induced ferroptosis disrupts the Th1/Th2 balance, skewing it towards a Th2-dominant response, which aligns with the immune profile observed in clinical RVVC patients. After Fer-1 intervention, the TNF-α/IL-4, IFN-γ/IL-4, and IL-2/IL-4 ratios increased significantly (*p* < 0.05). This demonstrates that inhibiting ferroptosis can restore Th1/Th2 immune balance by modulating the relative expression of Th1 and Th2 cytokines.

In summary, the results from this section confirm that ferroptosis activation in the vaginal tissue of CVVC mice promotes disease progression by exacerbating mucosal structural damage, disrupting the cytokine balance, and disturbing Th1/Th2 immune homeostasis. Conversely, Fer-1, by inhibiting ferroptosis, improves the local vaginal microenvironment through a multi-faceted approach: repairing the tissue barrier, mitigating cellular damage, regulating inflammatory cytokines, and restoring immune balance. This provides multi-dimensional experimental evidence supporting the potential of targeting ferroptosis for RVVC intervention.

## 4. Discussion

RVVC is a challenging mucosal disorder marked by chronic inflammation, persistent *Candida* colonization, and impaired local immune homeostasis. Despite antifungal therapy, relapse remains common, underscoring the need to clarify upstream tissue-injury programs and therapeutically tractable pathways [49]. Here, by integrating patient transcriptomics with in vivo CVVC modeling and in vitro macrophage infection assays, we provide convergent evidence that ferroptosis is functionally relevant to RVVC pathology. Rather than proposing ferroptosis as a singular initiating event, our data support a model in which ferroptosis interacts with established pathogenic processes—oxidative stress, epithelial barrier disruption, immune dysregulation, and fungal virulence-associated injury—to amplify inflammation and compromise antifungal effector function. Targeting ferroptosis may therefore help dampen the feed-forward loop of oxidative injury and immune imbalance that favors persistence and recurrence.

### 4.1. Ferroptosis-Related Signatures in RVVC and Pathway-Level Convergence

Intersecting RVVC DEGs with curated ferroptosis genes identified 194 shared targets enriched for ferroptosis regulators as well as inflammatory and immune mediators. Pathway analyses consistently highlighted ferroptosis together with IL-17 and HIF-1 signaling, two pathways tightly linked to *Candida*-driven mucosal inflammation and hypoxic/iron-related stress responses [50,51]. This convergence suggests that ferroptosis-associated programs may couple dysregulated redox/iron homeostasis with inflammatory signaling in the vaginal microenvironment.

### 4.2. Ferroptosis and Immune Dysregulation Beyond Macrophages

RVVC is widely regarded as an immunopathology-dominant mucosal disease in which clinical symptoms primarily arise from dysregulated host immune responses rather than uncontrolled fungal proliferation per se [52]. Consistent with this paradigm, immune deconvolution analysis in our study revealed a pro-inflammatory immune landscape characterized by increased M1 macrophages and activated CD4^+^ T cells, accompanied by reductions in regulatory or protective adaptive immune populations. The strong association between ferroptosis-related gene signatures and this skewed immune profile suggests that ferroptosis may contribute to immune remodeling within the vaginal microenvironment.

Although our mechanistic investigations focused primarily on macrophages, ferroptosis-associated immune perturbations are unlikely to be restricted to a single cell type. Neutrophils, which are abundant in the vaginal lumen during VVC/RVVC, generate high levels of reactive oxygen species and lipid oxidants and have been shown to undergo or regulate ferroptosis-like programs under inflammatory conditions [53]. In parallel, emerging evidence indicates that ferroptosis in dendritic cells can impair antigen presentation and disrupt immune regulation, thereby altering downstream T-cell responses [54]. Importantly, ferroptotic cell death is increasingly recognized as immunologically active rather than silent: ferroptotic cells can release damage-associated molecular patterns (DAMPs) and oxidized lipid mediators that further recruit and activate innate immune cells, amplifying inflammatory signaling and sustaining immune dysregulation [55].

Together, these observations support a model in which ferroptosis functions as an inflammatory amplifier in RVVC. Persistent fungal stimulation and oxidative stress may induce ferroptosis in epithelial and myeloid compartments, leading to DAMP release and lipid peroxidation-driven immune activation. This process may reinforce a feed-forward loop of inflammation that is robust yet poorly protective, ultimately favoring tissue damage and recurrence rather than effective antifungal immunity.

### 4.3. Macrophage Ferroptosis as a Functional Link to Impaired Antifungal Defense

Our in vivo and in vitro data identify macrophages as a prominent cellular target of ferroptosis during *C. albicans* exposure. Infection was associated with lipid peroxidation and iron/antioxidant imbalance (lipid ROS, 4-HNE, ACSL4, Fe^2+^, MDA increases; GPX4/SLC7A11/GSH decreases) and mitochondrial injury—features consistent with ferroptotic stress—together with reduced macrophage viability and antifungal killing capacity. Importantly, ferroptosis inhibition with Fer-1 partially restored macrophage survival and function, reduced fungal burden, and alleviated tissue injury, supporting macrophage ferroptosis as a mechanistically plausible contributor to persistence and immunopathology.

Beyond macrophages, vaginal epithelial cells are central to barrier integrity and inflammatory signaling in VVC/RVVC [56,57]. Our tissue-level observations (e.g., increased 4-HNE deposition and reduced GPX4 signal within infected mucosa) are compatible with ferroptosis-associated oxidative injury extending to epithelial compartments, potentially linking ferroptotic stress to barrier disruption and inflammatory amplification [13,56,57].

### 4.4. Ferroptosis Disrupts Mucosal Integrity and Immune Homeostasis

Histopathological and biochemical assessments demonstrated that ferroptosis-associated oxidative injury likely contributes to vaginal epithelial damage as indicated by increased LDH release, loss of epithelial integrity, and heightened inflammatory infiltration.

Furthermore, ferroptotic perturbation skews the local cytokine milieu towards a Th2-dominant response. Fer-1 treatment reversed these changes. Although Fer-1 reduced both Th1- and Th2-associated cytokines, this pattern is unlikely to represent simple immunosuppression. Instead, our results suggest that Fer-1 promotes immune normalization, in which excessive inflammatory polarization is attenuated while mucosal homeostasis is restored. In the context of RVVC, inflammatory pathology may be sustained by oxidative stress and epithelial injury, leading to a cytokine milieu that is amplified but not necessarily protective. By limiting ferroptosis-driven lipid peroxidation, Fer-1 may decrease damage-associated inflammatory amplification and re-establish a more balanced immune set-point. Notably, the concurrent improvement in fungal burden, tissue histopathology and epithelial injury markers argues against immune paralysis and supports the clinical relevance of restoring immune proportionality rather than dampening antifungal defense.

### 4.5. Therapeutic Implications: Targeting Ferroptosis in RVVC

Our study provides preclinical evidence that inhibiting ferroptosis with Fer-1 can alleviate vaginal tissue damage, reduce fungal burden, and rebalance the immune response. These effects are mediated through multiple mechanisms: reducing lipid peroxidation, restoring GPX4 and SLC7A11 expression, modulating iron homeostasis, and suppressing inflammatory cascades. Considering the limitations of current antifungal regimens—which often fail to address underlying inflammation and immune dysfunction—ferroptosis inhibitors represent a novel strategy to break the cycle of recurrence by targeting both cell death and immune dysregulation.

### 4.6. Limitations and Future Directions

While our study supports a strong association and functional involvement of ferroptosis-related pathways in RVVC, several limitations and open questions remain. Our mechanistic interpretation is based on convergent biochemical, molecular, morphological/ultrastructural, and pharmacological evidence, yet it relies largely on Fer-1 responsiveness. We acknowledge that Fer-1 has antioxidant actions beyond ferroptosis inhibition; however, in our system its protective effects were consistently accompanied by reversal of canonical ferroptotic features (e.g., restoration of GPX4/SLC7A11 and reduction in lipid ROS and ACSL4), arguing against nonspecific anti-inflammatory effects alone. Nonetheless, pharmacologic evidence cannot fully establish pathway specificity. Future studies should incorporate orthogonal ferroptosis modulators and genetic perturbation of key regulators (e.g., GPX4, SLC7A11, ACSL4), ideally using conditional and tissue-/cell type-specific approaches, to refine causality and compartmental contributions. Because infectious inflammation can engage multiple regulated cell death programs [58], and ferroptosis is typically defined by integrated biochemical/morphological/pharmacological criteria rather than exclusive elimination of alternative pathways, we view ferroptosis as a functional contributor rather than the sole initiating mechanism in RVVC. Consistently, we did not comprehensively profile apoptosis, necrosis, or pyroptosis; systematic cell death profiling and targeted perturbations are needed to define crosstalk and temporal dynamics. Moreover, upstream triggers of ferroptosis in the vaginal niche (fungal factors, host metabolites, and/or dysbiosis) remain to be clarified, and time-resolved, cell type-specific studies will be required to place ferroptosis relative to epithelial barrier disruption, cytokine-driven immune remodeling (Th1/Th2/Th17 balance), and virulence-associated tissue injury.

Model- and translation-related considerations also apply. Although the estrogen-dependent CVVC model reproducibly captures persistent colonization and chronic inflammation, it does not fully mirror episodic relapse cycles; recurrent challenge designs may better approximate clinical RVVC but are less standardized and may increase variability, warranting further validation. While ATCC 10231 provides a standardized platform, clinical isolates may show strain-specific virulence that modulates epithelial injury and immune responses, supporting replication across diverse strains. Methodologically, the uninfected controls did not receive vehicle injections, so procedural effects cannot be completely excluded. Translationally, the human vaginal ecosystem (microbiome complexity, pH, metabolic substrates) and hormonal status—particularly estrogen—may alter oxidative stress, lipid peroxidation kinetics, and iron handling, and should be considered in future studies [59,60]. Finally, because ROS/lipid peroxidation also support antimicrobial defense, immune signaling, tissue repair, and epithelial turnover, safety is critical: excessive or systemic ferroptosis inhibition could compromise ROS-dependent antifungal functions, perturb host–microbe homeostasis, or affect mucosal renewal, and inhibitors may have off-target antioxidant effects. Accordingly, future work should evaluate dose–response, infection clearance, systemic immune competence, and long-term mucosal integrity, and explore local delivery or cell type-targeted strategies; ultimately, clinical translation will require careful safety and efficacy testing in humans.

## 5. Conclusions

**Figure 18:**
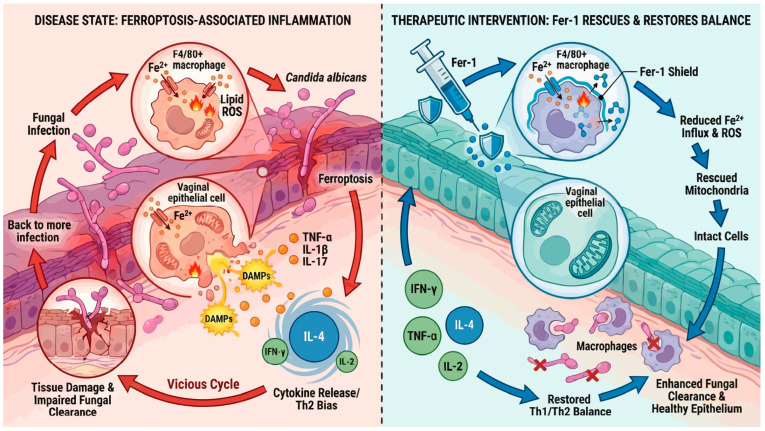
Proposed model of ferroptosis–inflammation–immunity coupling.

In summary, by integrating patient transcriptomic analyses with in vivo and in vitro validation, our study supports a functional association between RVVC and ferroptosis-related pathways. The data suggest that ferroptotic stress in vaginal tissues—particularly within macrophage populations—may contribute to inflammatory amplification, impaired antifungal effector function, and mucosal injury. Pharmacological inhibition of ferroptosis with Fer-1 alleviated tissue damage, improved immune balance, and reduced fungal burden in a CVVC model, providing proof-of-concept that ferroptosis modulation could complement current antifungal strategies. A schematic model summarizing the proposed “ferroptosis–inflammation–immunity” axis in RVVC is provided in Figure 18.

## Figures and Tables

**Table 1:** Top 20 in network by Betweenness method.

Rank	Name	Score
1	TNF	4917
2	IL6	3287
3	IL1B	3051
4	STAT3	2431
5	PTGS2	1945
6	TFRC	1556
7	APP	1540
8	HMOX1	1241
9	ALOX5	1023
10	SLC7A11	1010
11	CDK1	783
12	CDKN2A	743
13	EPAS1	718
14	BRCA1	681
15	NLRP3	666
16	IFNG	648
17	EIF2AK2	629
18	ACSL4	626
19	NEDD4	602
20	CXCL8	595

## Data Availability

The original contributions presented in this study are included in the article/Supplementary Materials. Further inquiries can be directed to the corresponding author.

## Supplementary Materials

The following supporting information can be downloaded at: https://www.mdpi.com/article/10.3390/antiox15040407/s1, Figure S1: Establishment of a murine model of CVVC; Figure S2: Validation of BMDMs purity; Figure S3: Fer-1 inhibits *C. albicans*-induced lipid ROS accumulation in BMDMs; Figure S4: Fer-1 restores MMP in *C. albicans*-infected BMDMs; Table S1: Gene information of FerrDB; Table S2: Comparative analysis of significantly DEPs in vaginal tissues between the control and the *C.albicans* group; Table S3: Comparative analysis of significantly DEPs in vaginal tissues between the *C.albicans* infection group and the *C.albicans*+Fer-1; Table S4: The effect of inhibiting ferroptosis on the levels of TNF-α, IFN-γ, IL-2 and IL-4 in vaginal lavage fluid of CVVC mice and their ratios to IL-4.

## Abbreviations

The following abbreviations are used in this manuscript:

RVVC	Recurrent Vulvovaginal Candidiasis
GEO	Gene Expression Omnibus
CVVC	Chronic Vulvovaginal Candidiasis
BMDMs	Bone Marrow-derived Macrophages
DEGs	Differentially Expressed Genes
PPI	Protein-protein Interaction
GSEA	Gene Set Enrichment Analysis
GO	Gene Ontology
KEGG	Kyoto Encyclopedia of Genes and Genomes
VVC	Vulvovaginal Candidiasis
CTRL	Healthy Controls
SPF	Specific Pathogen-free
SDA	Sabouraud Dextrose Agar
SDB	Sabouraud’s Dextrose Broth
PBS	Phosphate-buffered Saline
CFU	Colony-forming Units
GMS	Grocott’s methenamine silver
MMP	Mitochondrial Membrane Potential
TEM	Transmission Electron Microscopy
SD	Standard Deviation
LDH	Lactate Dehydrogenase
DEPs	Differentially Expressed Proteins
