# Supplementary material for: Ferroptosis in Recurrent Vulvovaginal Candidiasis Through Integrated Bioinformatics and Experimental Validation
Source: Antioxidants (Basel). 2026 Mar 24;15(4):407. doi: 10.3390/antiox15040407 (PMC13113882; doi:10.3390/antiox15040407)
Supplement: Supplementary file 1 [file antioxidants-15-00407-s001.zip › antioxidants-4098603-supplementary.pdf]

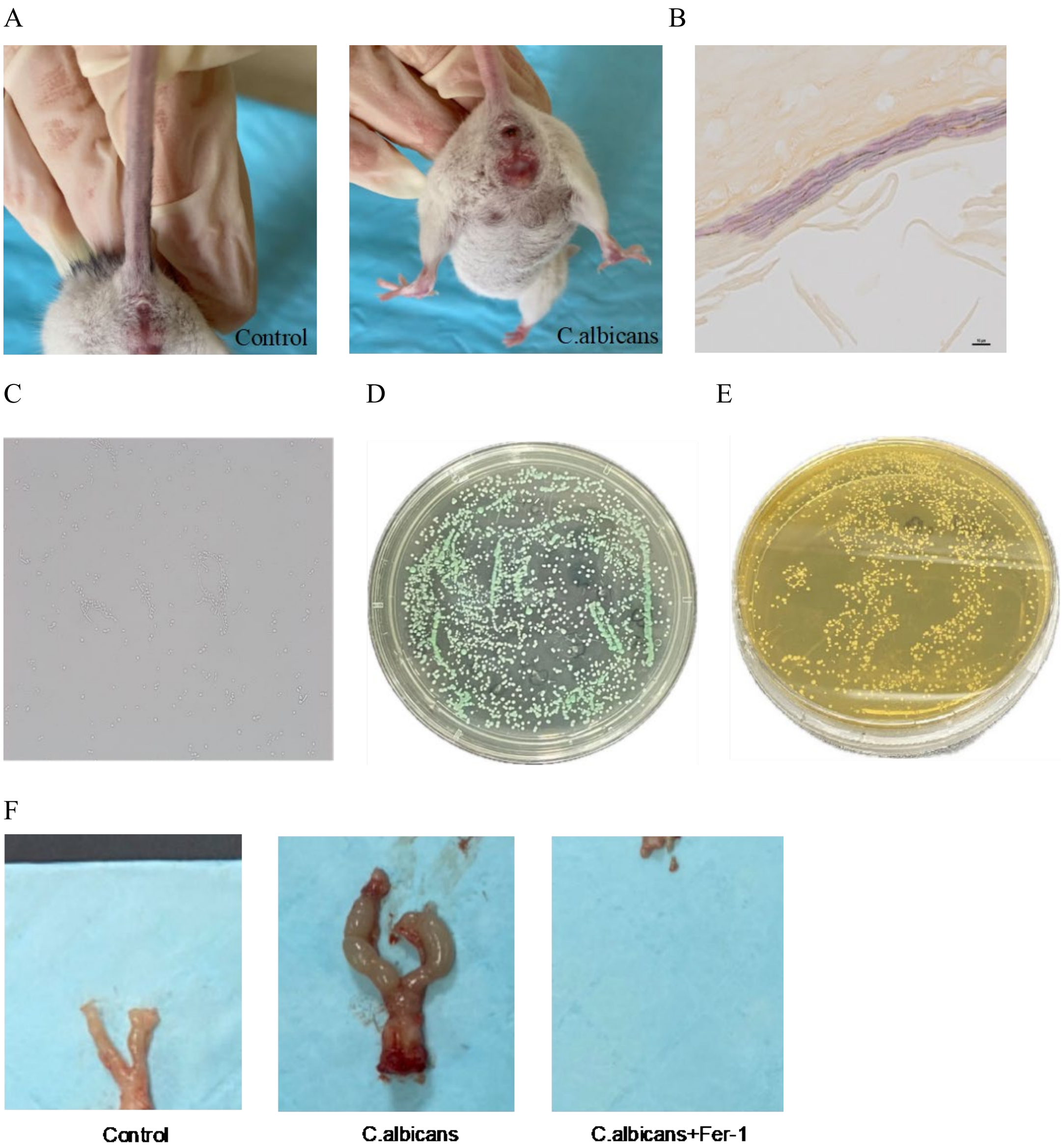

Supplementary Figure S1 Establishment of a murine model of CVVC.

(A) Vulvovaginal appearance of mice in the control group and the C.albicans infection group. (B) Vaginal tissue from the C.albicans infection group stained with hexamine silver (bar = 10 μm, ×400). (C) Optical microscopy of vaginal lavage fluid from the C.albicans infection group (×200). (D) Chromogenic agar plate culture of vaginal lavage fluid from the C.albicans infection group. (E) Sabouraud dextrose agar culture of vaginal lavage fluid from the C.albicans infection group. (F) Comparison of gross morphology of vaginal tissues among the control, the C.albicans infection group, and the C.albicans+Fer-1 group.

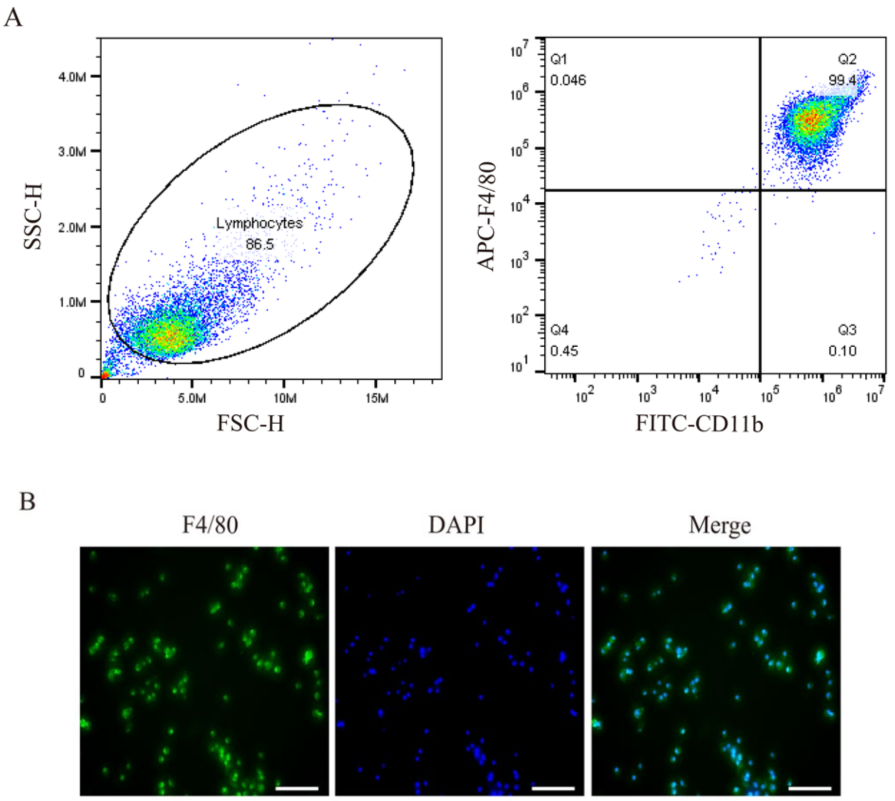

Supplementary Figure S2. Validation of BMDMs purity

(A) Flow cytometric analysis of isolated BMDMs. Representative dot plots show dual positivity for the macrophage-specific surface markers F4/80 and CD11b, with over 98% of cells falling within the double-positive quadrant, confirming high purity.(B) IF staining of BMDMs: F4/80 (green, macrophage marker) + DAPI (blue, nuclei); Merge panel confirms F4/80 expression in BMDMs. (bar 5 μm, ×200; n = 3 independent isolations).

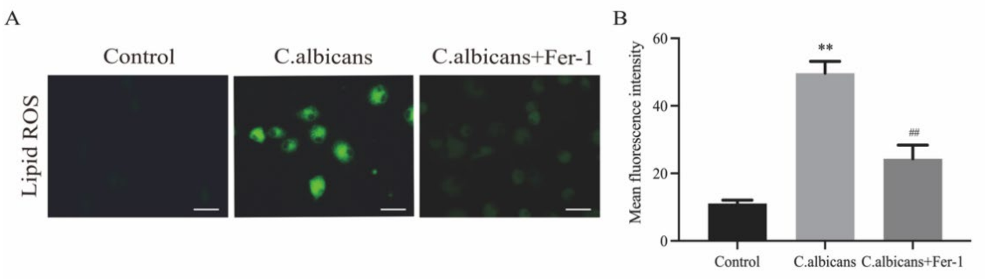

Supplementary Figure S3. Fer-1 inhibits C. albicans-induced lipid ROS accumulation in BMDMs

(A) IF staining of lipid ROS (green) in BMDMs using BODIPY 581/591 C11 probe. bar 5 μm, ×200. (B) Quantitative analysis of lipid ROS MFI from IF staining. Control (control group), C. albicans (C. albicans -infected BMDMs

group), C. albicans+Fer-1 (C. albicans -infected BMDMs group +Fer-1); Data are mean ± SD (n=3 independent experiments); *P < 0.05, **P < 0.01 vs. Control; ##P < 0.01 vs. C. albicans group.

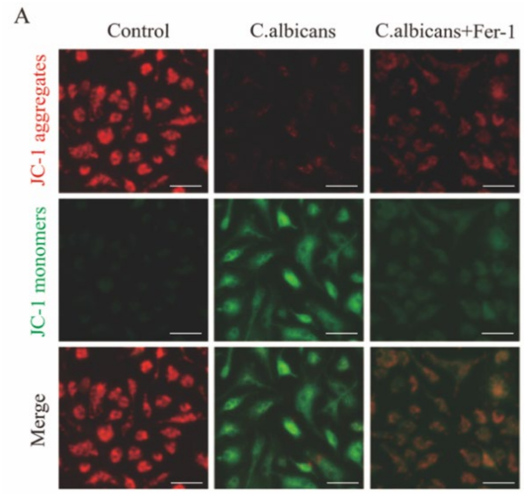

Supplementary Figure S4. Fer-1 restores MMP in C. albicans-infected BMDMs

IF staining of MMP (JC-1 probe): Red = JC-1 aggregates (high MMP); Green = JC-1 monomers (low MMP); Merge panels show MMP status across groups. bar 5 μm, ×200. groups: Control (control group), *C. albicans* (*C. albicans* -infected BMDMs group), *C. albicans*+Fer-1 (*C. albicans* -infected BMDMs group +Fer-1); (Representative images from **3 independent experiments**).

Supplementary Table S2 Comparative analysis of significantly DEPs in vaginal tissues between the control and the *C.albicans* group

DEPs	AveExp. Control	AveExp. C.albicans	logFC	fold change	regulation
TECK	10.33404977	9.902958542	-0.431091225	0.741700566	down
IL-3	9.72417275	9.366924571	-0.357248179	0.78065219	down
KC	11.17636018	10.85838314	-0.317977038	0.802193934	down
Eotaxin	10.54428831	10.27384278	-0.270445531	0.829063476	down
I-TAC	12.64358566	12.97437576	0.330790096	1.25770197	up
TIMP-2	10.67666233	11.04442869	0.367766355	1.2903535	up
TIMP-1	8.295998462	8.671626635	0.375628173	1.297404343	up
S100A8	8.684047262	9.066018899	0.381971637	1.303121531	up
sTNF RI	13.50081699	13.88392151	0.383104521	1.304145215	up
MCP-1	12.87186674	13.29628042	0.424413675	1.342026979	up
IFN-γ	9.514319552	9.939051826	0.424732274	1.34232338	up

TNF-α	11.64849241	12.34912321	0.7006308	1.625215242	up
MIP-1α	13.50597278	14.21540287	0.709430085	1.635158045	up
IL-2	11.2643253	12.09379295	0.829467651	1.777029524	up
RANTES	8.070389328	8.918866319	0.848476991	1.800599084	up
IL-1β	10.16465556	11.04944932	0.884793759	1.846500636	up
G-CSF	11.50422312	12.59607007	1.091846954	2.131467347	up
IL-17	7.699051844	8.922734106	1.223682262	2.335420381	up
IL-4	10.26830806	12.48731781	2.21900975	4.655737602	up

Supplementary Table S3 Comparative analysis of significantly DEPs in vaginal tissues between the *C.albicans* infection group and the *C.albicans*+Fer-1

DEPs	AveExp. C.albicans	AveExp. C.albicans+Fer-1	logFC	fold change	regulation
IL-4	12.48731781	10.247413	-2.239904817	0.211700295	down
RANTES	8.918866319	7.487918476	-1.430947843	0.370887142	down
IL-2	12.09379295	10.92625127	-1.167541685	0.445179269	down
TIMP-2	11.04442869	10.15840798	-0.886020705	0.541104556	down
TNF-α	12.34912321	11.47741551	-0.871707693	0.546499586	down
G-CSF	12.59607007	11.75994393	-0.836126143	0.560145628	down
IFN-γ	9.939051826	9.197431975	-0.74161985	0.598067468	down
S100A8	9.066018899	8.359584194	-0.706434705	0.612832747	down
SDF-1	10.12791185	9.425061489	-0.702850364	0.614357208	down
IL-17	8.922734106	8.236624387	-0.686109718	0.621527565	down
IL-1β	11.04944932	10.37246024	-0.67698908	0.625469274	down
IL-1α	11.22207401	10.54761606	-0.674457947	0.62656759	down
M-CSF	12.39148901	11.74873762	-0.642751394	0.640490291	down
sTNF RII	11.83265875	11.21058109	-0.622077657	0.649734556	down
MCP-1	13.29628042	12.75884572	-0.537434696	0.688994945	down
LIX	13.19277511	12.74697803	-0.445797088	0.734178572	down
TIMP-1	8.671626635	8.249587172	-0.422039463	0.746368775	down
TCA-3	12.48415855	12.06240613	-0.421752413	0.746517294	down
FAS ligand	10.36621043	9.974891696	-0.391318738	0.762432362	down
IL-13	11.07139295	10.68278087	-0.388612078	0.763864114	down

Eotaxin-2	10.97844825	10.69162851	-0.286819741	0.81970702	down
I-TAC	12.97437576	12.71052659	-0.263849161	0.832862845	down
IL-12	9.308011518	9.792688355	0.484676837	1.399272394	up

Supplementary Table S4 The effect of inhibiting ferroptosis on the levels of TNF-α, IFN-γ, IL-2 and IL-4 in vaginal lavage fluid of CVVC mice and their ratios to IL-4

Group	Control	*C. albicans*	*C. albicans* + Fer-1
TNF-α (ng/L)	160.94±19.95	503.55±48.74	299.09±31.26
IFN-γ (pg/mL)	67.23±6.42	148.76±48.74	134.42±8.69
IL-2 (pg/mL)	27.29±5.38	65.57±6.96	40.76±6.75
IL-4 (pg/mL)	11.06±1.22	80.13±6.09	30.22±6.57
TNF-α/IL-4	14.80±2.95	6.31±0.73**	10.22±1.91##
IFN-γ/IL-4	6.15±0.88	1.86±0.20**	4.64±1.08##
IL-2/IL-4	2.51±0.64	0.82±0.99**	1.41±0.37##

*P < 0.05 vs. Control; **P < 0.01 vs. Control; #P < 0.05 vs. *C. albicans* group ; ##P < 0.01 vs. *C. albicans* group; Mice were treated as follows: Control (control group), *C. albicans* (*C. albicans* -infected group), *C. albicans*+Fer-1 (*C. albicans* -infected group +Fer-1)..
